# Mutant Isocitrate Dehydrogenase Inhibitors as Targeted Cancer Therapeutics

**DOI:** 10.3389/fonc.2019.00417

**Published:** 2019-05-17

**Authors:** Danielle Golub, Nishanth Iyengar, Siddhant Dogra, Taylor Wong, Devin Bready, Karen Tang, Aram S. Modrek, Dimitris G. Placantonakis

**Affiliations:** ^1^Department of Neurosurgery, New York University School of Medicine, NYU Langone Health, New York, NY, United States; ^2^Clinical and Translational Science Institute, New York University School of Medicine, NYU Langone Health, New York, NY, United States; ^3^New York University School of Medicine, NYU Langone Health, New York, NY, United States; ^4^Division of Hematology/Oncology, Department of Pediatrics, New York University School of Medicine, NYU Langone Health, New York, NY, United States; ^5^Department of Radiation Oncology, New York University School of Medicine, NYU Langone Health, New York, NY, United States; ^6^Kimmel Center for Stem Cell Biology, New York University School of Medicine, NYU Langone Health, New York, NY, United States; ^7^Laura and Isaac Perlmutter Cancer Center, New York University School of Medicine, NYU Langone Health, New York, NY, United States; ^8^Brain Tumor Center, New York University School of Medicine, NYU Langone Health, New York, NY, United States; ^9^Neuroscience Institute, New York University School of Medicine, NYU Langone Health, New York, NY, United States

**Keywords:** acute myeloid leukemia, enasidenib, glioma, IDH, isocitrate dehydrogenase, ivosidenib

## Abstract

The identification of heterozygous neomorphic isocitrate dehydrogenase (IDH) mutations across multiple cancer types including both solid and hematologic malignancies has revolutionized our understanding of oncogenesis in these malignancies and the potential for targeted therapeutics using small molecule inhibitors. The neomorphic mutation in IDH generates an oncometabolite product, 2-hydroxyglutarate (2HG), which has been linked to the disruption of metabolic and epigenetic mechanisms responsible for cellular differentiation and is likely an early and critical contributor to oncogenesis. In the past 2 years, two mutant IDH (mutIDH) inhibitors, Enasidenib (AG-221), and Ivosidenib (AG-120), have been FDA-approved for IDH-mutant relapsed or refractory acute myeloid leukemia (AML) based on phase 1 safety and efficacy data and continue to be studied in trials in hematologic malignancies, as well as in glioma, cholangiocarcinoma, and chondrosarcoma. In this review, we will summarize the molecular pathways and oncogenic consequences associated with mutIDH with a particular emphasis on glioma and AML, and systematically review the development and preclinical testing of mutIDH inhibitors. Existing clinical data in both hematologic and solid tumors will likewise be reviewed followed by a discussion on the potential limitations of mutIDH inhibitor monotherapy and potential routes for treatment optimization using combination therapy.

## Introduction

The discovery of mutations in isocitrate dehydrogenase 1 (IDH1) and 2 (IDH2) in over 80% of low-grade gliomas (LGGs) and secondary glioblastomas has revolutionized pharmaceutical approaches to targeted therapies and the overall glioma classification schema (1, 2). Driver mutations in IDH1 and IDH2 have been likewise identified in acute myeloid leukemia (AML), chondrosarcoma, myelodysplastic syndromes, and cholangiocarcinoma (3–6). Limitations in current treatment options, particularly in LGG and AML, due to both inefficacy and systemic toxicity, make mutant IDH (mutIDH), and its associated molecular pathways attractive therapeutic targets (7–9). Major strides in developing and testing candidates for mutIDH inhibition have been made in the past few years with the FDA approvals of Ivosidenib (Tibsovo®) and Enasidenib (Idhifa®), selective mutIDH1 and mutIDH2 inhibitors, respectively (10, 11). While these agents have had some preliminary success in AML, utility in the treatment of IDH-mutant glioma or other IDH-mutated cancers has not been established (12, 13).

IDH1 and IDH2 are homodimeric isoenzymes involved in a major pathway for cellular NADPH generation through the oxidative decarboxylation of isocitrate to α-ketoglutarate. IDH1 is found in the cytosol and in peroxisomes, while IDH2 is a mitochondrial enzyme. Mutations in IDH3 isoforms, which form heterotetrameric complexes in mitochondria, are rarely seen in cancer, but there is some evidence that upregulation of wild-type IDH3 may contribute to various tumorigenic metabolic pathways (14, 15). The IDH1/2 mutations are heterozygous and neomorphic in that they establish a pathway for the NADPH-dependent conversion of the wild-type IDH product, α-ketoglutarate, to 2-hydroxyglutarate (2HG) (16). Simultaneously, significant decreases in NADPH production are also seen (17). Early structural and pharmacokinetic studies show that mutant IDH develops an increased affinity for both the cofactor NADPH and substrate α-ketoglutarate (16, 18). In the most common IDH1/2 mutants, the wild-type IDH function of oxidative decarboxylation of isocitrate to α-ketoglutarate is lost due to mutation of critical amino acid residues in the catalytic domain, IDH1 R132 and IDH2 R172, which are normally responsible for binding the β-carboxyl group of isocitrate and initiating catalysis (1, 16, 18). Interestingly, there is some evidence that, unlike the IDH1 mutant, the IDH2 mutant may not depend on heterodimerization with an IDH wild-type partner for 2HG production (19). Nevertheless, while the mutant IDH enzyme can exist either as a homodimer or as a heterodimer with the wild-type IDH within cancer cells, all reported oncogenic IDH mutations to date are genetically heterozygous, suggesting that the critical role of mutant IDH is related to its gain-of-function for conversion of the wild-type IDH product, α-ketoglutarate, to 2HG (20).

**Figure 1:**
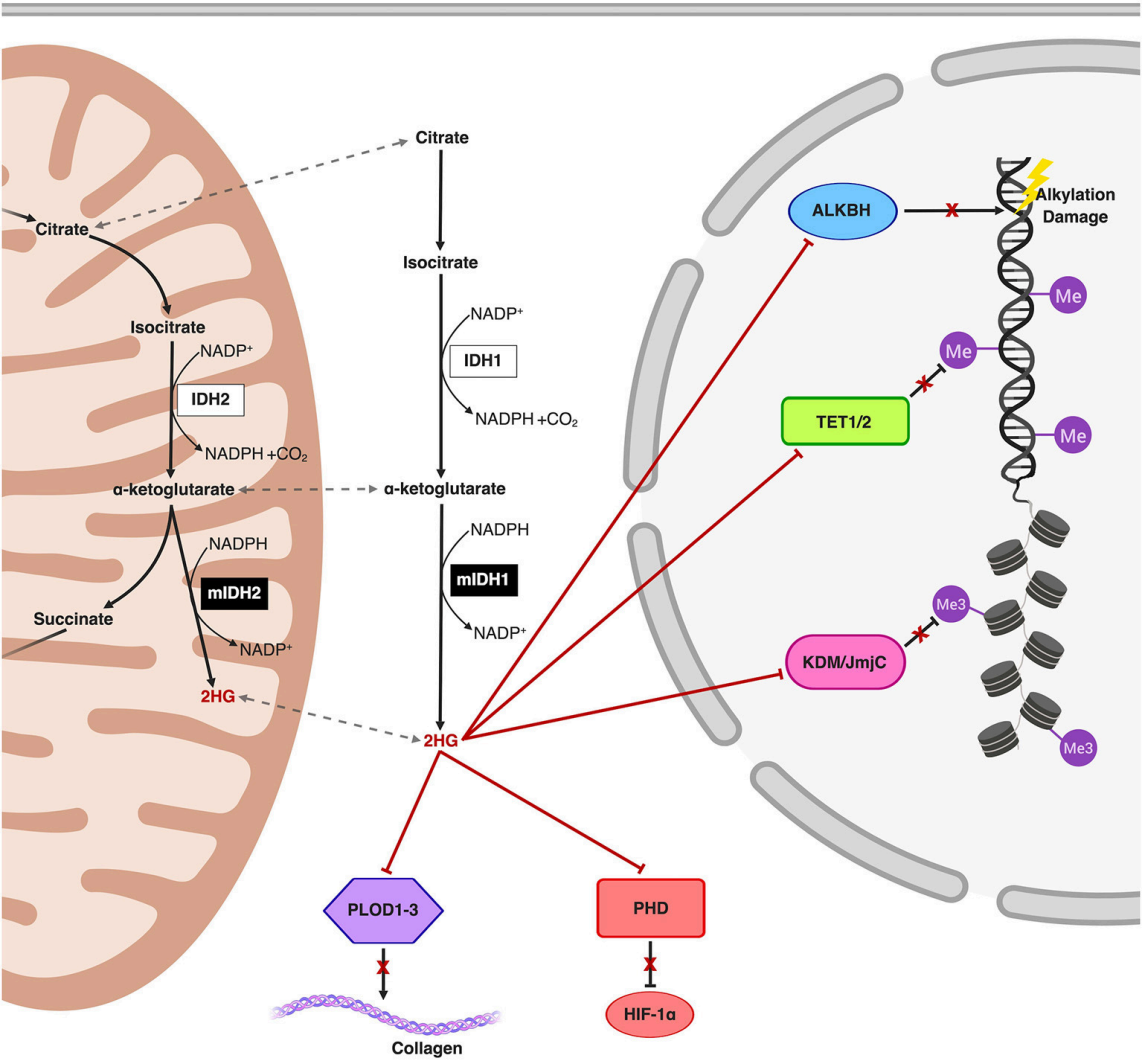
Schematic representation of the mutIDH1 and mutIDH2 pathways and molecular mechanisms related to oncogenesis. The neomorphic enzyme, mutIDH1/2, converts the wild-type IDH product, α-ketoglutarate, to the oncometabolite, 2-hydroxyglutarate (2HG) both in the cytosol and in the mitochondria. 2HG competitively inhibits α-ketoglutarate-dependent dioxygenases both in the cytosol and in the nucleus. 2HG-mediated inhibition of the activity of Ten-Eleven Translocation (TET) enzymes and histone lysine demethylases (KDM) result in global epigenetic modifications on DNA and histones, respectively, resulting in a hypermethylator phenotype. Inhibition of prolyl hydroxylases and lysyl hydroxylases (such as PLOD1-3) interferes with both collagen maturation and with the degradation pathway of Hypoxia-Inducible Factor 1α (HIF-1α), thereby post-translationally stabilizing HIF-1α. Additionally, ALKBH, responsible for repair of oxidative DNA damage, is also inhibited by 2HG, an effect which potentially introduces risk for increased mutational burden.

Accumulation of 2HG, increasingly well-characterized as an oncometabolite, disrupts multiple regulatory cellular pathways involving α-ketoglutarate-dependent dioxygenases including those involved in epigenetic remodeling and DNA repair (Figure 1) (21–23). Structural similarities between α-ketoglutarate and 2HG allow the latter to competitively occupy the same pockets as α-ketoglutarate in α-ketoglutarate-dependent dioxygenases (of which over 60 have been described in humans), without promoting enzymatic activation (22, 24–26). Changes in the epigenetic landscape brought on by 2HG-mediated disruption of the ten-eleven translocation (TET) family of 5-methylcytosine (5 mC) hydroxylases (DNA demethylases) and the JmJC domain-containing histone lysine demethylases (KDMs) are hypothesized to promote oncogenesis through DNA and histone hypermethylation and resultant transcriptional dysregulation (22, 27). The resulting global increase in DNA methylation in the mutIDH context is aptly named the CpG Island Methylator Phenotype (CIMP) (28, 29). Manipulating and reversing the oncogenic IDH-mutant methylome is the primary molecular endpoint for therapeutic IDH inhibition and 2HG reduction in both glioma and AML. It remains to be seen, however, if 2HG reduction alone will be sufficient to reverse oncogenic changes to the methylome, as epigenetic memory persists through daughter cells via methyltransferases, a topic we explore further in our discussion (30, 31).

Here, we provide an overview of the current literature on IDH mutations in cancer with a particular emphasis on glioma and AML and the potential for mutIDH as a therapeutic target in these contexts. We describe the current evidence for the various generations of mutIDH inhibitors through the drug-discovery, preclinical, and clinical stages and systematically review related past and ongoing clinical trials. We furthermore describe the possible adverse effects of IDH inhibitors, such as “differentiation syndrome,” and conclude with a discussion on the potential for enhancing the efficacy of IDH inhibitors in combination with epigenetic modification-based therapies.

### IDH Mutations in Glioma

Ten years ago, our understanding of the molecular landscape in glioma was transformed by the first genome-wide analysis of somatic mutations in glioblastoma (GBM) and the identification of recurrent mutations in IDH1 nearly exclusively in secondary GBM (2). Mutations in IDH1 and IDH2 are seen in over 80% of lower-grade gliomas (WHO grades II and III) and secondary GBMs that are thought to later develop from lower-grade lesions (2, 32, 33). The vast majority of somatic IDH mutations (>95%) are seen in IDH1, and the most commonly observed IDH1 mutation occurs at the R132 residue (1, 34). IDH2 mutations, which are mutually exclusive with those in IDH1 and found at a functionally analogous R172 residue, only represent a minority of somatic IDH mutations in glioma (35, 36).

IDH-mutant gliomas are generally further categorized into two major subtypes: those with chromosome 1p/19q co-deletion, historically termed oligodendrogliomas; and those without 1p/19q co-deletion, also known as astrocytomas (37). These two groups are biologically and clinically distinct. Up to 94% of IDH-mutant non-1p/19q co-deleted gliomas harbor loss-of-function TP53 mutations and 86% have inactivating ATRX mutations (37). Only few IDH mutant astrocytomas carry IDH wild-type driver mutations or copy number alterations, and those who do (for example CDKN2A or CDKN2B loss) are usually classified as IDH mutant GBM (1). These robust genomic differences are highly suggestive of a unique mechanism of oncogenesis in the IDH-mutant subgroup and furthermore imply that the IDH mutation is likely an early player in a cell-of-origin, which in its native state is capable of giving rise to both astrocyte and oligodendrocyte lineages. Clinically, IDH-mutant lesions present in a younger age group (median age in the fourth vs. the sixth decade of life), when compared to IDH-wild type gliomas (33). Furthermore, IDH mutations are well-known to be an independent favorable prognostic factor at all stages of glioma progression; for example, the median survival in IDH-mutant GBM is 31 months, over twice as long as the median 15-months survival in the wild-type counterpart (1).

Consistent with other IDH-mutant cancers, IDH-mutant glioma is characterized by high levels of 2HG and the resulting “CIMP” hypermethylator phenotype described previously. In glioma specifically, these genome-wide DNA methylation changes have been shown to establish “insulator dysfunction” or disruption of topologically-associated domains (TADs) and thereby directly influence key transcriptional regulatory pathways related to gliomagenesis (38, 39). As previously mentioned, analyses of clonality among glioma tumor samples suggests that the IDH mutation is a tumor-initiating event in a common progenitor cell, hypothesized by many to be derived from the subventricular zone stem cell niche (7, 40–42). Despite our enhanced understanding of the molecular pathogenesis of IDH-mutant glioma, however, effective treatments have yet to be developed and clinicians remain reliant on maximal safe surgical resection and various chemotherapeutic agents and radiation treatments to prolong survival (7). Furthermore, a unique characteristic of LGG is its diffuse and highly infiltrative phenotype, making surgical resection rarely curative in the long term. To compound the complexity of these tumors, and historically popular chemotherapeutic agents have been shown to induce hypermutant recurrent tumors (7). Recent efforts in developing small molecule inhibitors that target IDH mutation provide a new opportunity for progress in glioma treatment.

### IDH Mutations in AML

Around the same time as the identification of recurrent IDH mutations in glioma, Mardis et al. published the results of a landmark study in which they sought to pinpoint recurrent mutations in AML that may be associated with the pathogenesis of the malignancy (43). In this study, the investigators identified for the first time the presence of IDH1 mutations in AML (43). 8.5% of analyzed samples had an IDH1 mutation at the R132 residue (mutated to either cysteine, histidine, or serine), which is also the site of the overwhelming majority of somatic IDH mutations in glioma (1, 34). Shortly after the discovery of IDH1 mutations in AML, another landmark study reported the first case of IDH2-mutated AML, in which the R172 residue was mutated to lysine (18). Further investigation of AML DNA samples revealed the existence of several additional cases of AML where the IDH2^R172^ residue was mutated (18). Interestingly, this study also found that a majority of the analyzed samples had IDH2 mutations (compared to IDH1 mutations) (18). This is in stark contrast to glioma, where the majority of IDH mutations are in IDH1.

Nearly one in five cases of AML is IDH-mutant, with IDH2-mutant AML being more prevalent than IDH1-mutant AML (11–13, 44–50). The IDH2^R140^ mutation (in particular, the R140Q variant) is the most common, with the IDH1^R132^ and IDH2^R172^ mutations also appearing frequently in the literature (3, 12, 45, 46, 50–52). Other mutations include, but are not limited to, IDH1^V71^ and IDH1 SNP rs11554137, a GGC to GGT transversion at the glycine residue at codon position 105 with unknown significance (47, 48, 53). Clinical and pathologic characteristics associated with IDH-mutant AML include normal karyotype (intermediate-risk cytogenetics), increased patient age, elevated platelet count, increased bone marrow blast percentage at initial presentation, increased peripheral blast percentage, decreased absolute neutrophil count (especially in IDH1-mutant AML), and concurrent mutations such as NPM1 and FLT3-ITD (44–47, 54). IDH1 and IDH2 mutations in AML are mutually exclusive, as in glioma. Likewise, in AML, IDH mutations are almost entirely mutually exclusive with TET2 mutations, suggesting that, mechanistically, these genes aref both involved in DNA hypermethylation as a driver of leukemogenesis (3, 45–47, 54).

It has been suggested that testing AML patients for IDH mutation status is simple and should be performed universally; however, the relationship between IDH mutation status and prognosis is considerably less clear and more controversial in AML than it is in other cancers such as glioma (46, 55). Most studies of IDH-mutant AML have suggested that mutIDH either foreshadows an adverse prognosis (given an association with increased blast percentage and older age at diagnosis) or is of little prognostic value (45, 48, 52, 55). Reported 2–3-years overall survival in IDH-mutant AML ranges between 51 and 89% in the literature; discrepancies are thought to be related to differences in cohort age, but some authors also argue that different specific IDH mutations may carry varied prognostic implications (3, 44, 45, 47, 54, 56, 57). Interestingly, IDH mutation status may also be useful for the detection of residual disease and prognostication following treatment; several studies investigating the value of serum 2HG during remission in AML have found that elevated serum 2HG levels actually predict shortened overall survival (55, 58, 59).

While induction/consolidation chemotherapy has revolutionized AML treatment strategy in the past 20 years, this standard-of-care universal treatment has evolved minimally since its introduction and is often contraindicated in elderly or otherwise frail patients (44). Given our enhanced understanding of the molecular and genetic subtypes of AML and the potential for targeted treatment, manipulation of these markers with small molecules may provide significant benefit. Drugs targeted to the mutIDH isotypes are one such example; for almost a decade, mutIDH inhibitors have been a focus of laboratory and clinical research in AML with great recent success leading to two FDA approvals specifically for AML indications.

### Drug Development and Preclinical Studies

Multiple mutIDH inhibitors, including one pan-inhibitor and several specific to one mutIDH isoform, have been developed over the last several years. A handful of these are in use in clinical trials, but only two have been approved by the FDA; Enasidenib and Ivosidenib (10, 11). A detailed review of the structural and pharmacokinetic properties and relevant preclinical data for both FDA approved inhibitors will follow a brief discussion of other mutIDH inhibitors with demonstrated and repeated preclinical efficacy (Table 1).

**Table 1:** MutIDH inhibitors.

**Compound**	**Target**	**Mechanism**	**Susceptible mutations**	**IC_**50**_**	**T_**1/2**_**	**C_**max**_**	**Time to C_**max**_**	**Cl**	**AUC**	**Mode of elimination**	**Penetrates BBB**	**Metabolism**	**SPONSOR(S)**
Ivosidenib (AG-120) (10, 13, 68)	mutIDH1	Reversible, allosteric, competitive inhibitor via cofactor (Mg) binding site	IDH1^R132H^ IDH1^R132C^ IDH1^R132G^ IDH1^R132S^ IDH1^R132L^	Biochemical: IDH1^R132H^: 12 nM IDH1^R132C^: 13 nM IDH1^R132G^: 8 nM IDH1^R132S^: 12 nM IDH1^R132L^: 13 nM Cell-Based: U87 (IDH1^R132H^): 19 nM HT1080 (IDH1^R132C^): 8 nM COR-L105 (IDH1^R132C^): 15 nM HCCC-9810 (IDH1^R132S^): 12 nM	93 h	6,551 ng/mL	~3 h	4.3 L/h	117, 348 ng∙h/mL	77% fecal 17% renal	Yes (4.1% penetrance in rat model)	CYP3A4, *N*-dealkylation, hydrolytic pathways	Agios, Celgene
ENASIDENIB (AG-221)^1^ (1, 12, 51)	mutIDH2	Allosteric, non-competitive inhibition via stabilization of open, inactive enzyme dimer conformation (steric hindrance)	IDH2^R140Q^ IDH2^R172K^	Biochemical (at 1*6 h*): IDH2^R140Q^: 100 nM IDH2^R172K^: 400 nM WT/IDH2^R140Q^: 30 nM WT/IDH2^R172K^: 10 nM Cell-Based: HCT-116 (IDH2^R172K^): 530 nM TF-1 (IDH2^R140Q^): 20 nM TF-1 (IDH2 ^R172K^): 980 nM U87 (IDH2^R140Q^): 10 nM U87 (IDH2 ^R172K^): 1,590 nM	137 h	1,300 ng/mL	~4 h	0.74 L/h	–	89% fecal 11% renal	-	CYP1A2, CYP2C9 CYP2C19, CYP2D6, CYP3A4, UGT1A1, UGT2B7, UGT2B15	Agios, Celgene
AG-881 (61, 62, 69)	Pan-inhibitor	Allosteric, non-competitive inhibition via stabilization of open, inactive enzyme dimer conformation (steric hindrance)	IDH1^R132H^ IDH1^R132C^ IDH1^R132G^ IDH1^R132S^ IDH1^R132L^ IDH2^R140Q^ IDH2^R172K^	Biochemical: IDH1^R132H^: 6 nM IDH1^R132C^: 19 nM IDH1^R132G^: 17 nM IDH1^R132S^: 6 nM IDH1^R132L^: 34 nM WT/ IDH1^R132H^: 4 nM IDH2^R140Q^: 12 nM (16 h) IDH2^R172K^: 94 nM (16 h) WT/IDH2^R140Q^: 32 nM (16 h) WT/IDH2^R172K^: 8 nM (16 h) Cell-Based: HCT-116 (IDH1^R132C^): 22nMHCT-116 (IDH1^R132H^): 3nMHCT-116 (IDH2 ^R172K^): 130nMCOR-L105 (IDH1^R132C^): 3.8nMHCC-9810 (IDH1^R132S^) 0.85nMHT1080 (IDH1^R132C^): 4nMJJ012 (IDH1^R132G^): 6.6nMTF-1 (IDH1^R132C^): 3.2nMTF-1 (IDH2^R140Q^): 14nMU87 (IDH2 ^R140Q^): 7.1nM	67.2 h	–	–	–	2,746 ng∙h/mL at 100 mg daily dose 7,020 ng∙h/mL at 200 mg daily dose	–	Yes (brain-to-plasma ratio 0.62-1.96 in mouse model, 1.11-1.48 in rat model)	–	Agios, Celgene
BAY-1436032 (52, 63)	mutIDH1	Allosteric, non-competitive inhibition via stabilization of open, inactive enzyme dimer conformation (steric hindrance)	IDH1^R132H^ IDH1^R132C^ IDH1^R132G^ IDH1^R132S^ IDH1^R132L^	Biochemical: IDH1^R132H^: 15 nM IDH1^R132C^: 15 nM Cell-Based: LN299 (IDH1^R123H^): 73 nM HCT-116 (IDH1^R132H^): 47 nM HT1080 (IDH1^R132C^): 135 nM NCH551b (GBM) (IDH1^R132H^): 13 nM AML (patient-derived)IDH ^R123H^: 5 nM IDH ^R123C^: 5 nM IDH ^R123G^: 4 nM IDH ^R123S^: 16 nM IDH ^R123L^: 3 nM	3.1 h (in rat model)	–	–	0.15 L/h/kg (in rat model)	–	–	Yes (brain-to-plasma ratio 0.08–0.38 in mouse model)	–	Bayer
AGI-5198 (66, 67, 70)	mutIDH1	Reversible, allosteric, competitive inhibitor to alpha-KG at the substrate binding site	IDH1^R132H^ IDH1^R132C^	Biochemical: IDH1^R132H^: 70 nM IDH1^R132C^: 160 nM Cell-Based: U87 (IDH1^R132H^): 70 nM HT1080 (IDH1^R132C^): 480 nM	–	–	–	–	208,000 ng∙h/mL (in mouse model via i.p. dosing)	-	Yes (shown to accumulate in mouse glioma xenografts)	–	Agios, Celgene
IDH305 (71)	mutIDH1	Allosteric, non-competitive inhibition via stabilization of open, inactive enzyme dimer conformation (steric hindrance)	IDH1^R132H^ IDH1^R132C^	Biochemical: IDH1^R132H^: 27 nM IDH1^R132C^: 28 nM Cell-Based: HCT-116 (IDH1^R132H^): 24 nM MCF10A (IDH1^R132H^): 20 nM	–	–	–	–	–	–	Yes (brain-to-plasma ratio 0.29–0.61 in murine models)	–	Novartis
AGI-6780 (72–74)	mutiDH2	Slow-tight binder, allosteric, non-competitive inhibition via stabilization of open, inactive enzyme dimer conformation (steric hindrance)	IDH2^R140Q^	Biochemical (at 1*6 h*): IDH2^R140Q^: 23 nM IDH2^R140Q^/WT: 4 nM Cell-Based: TF-1 (IDH2^R140Q^): 18 nM U87 (IDH2^R140Q^): 11 nM	–	–	–	–	–	-	–	–	Agios, Celgene
FT-2102 (75)	mutIDH1	–	–	–	60 h	–	–	–	–	–	–	–	Forma
HMS-101 (76, 77)	mutIDH1	Competitive inhibition, binds isocitrate-binding pocket	IDH1^R132C^	Cell-Based: HOXA9 (mouse bone marrow) (IDH1^R132C^): 1000 nM	–	–	–	–	–	–	–	–	Hannover Medical School (Germany)
MRK-A (78)	mutIDH1	–	IDH1^R132H^ IDH1^R132C^	Biochemical: IDH1^R132H^: 5 nM Cell-Based: MOG-G-UVW (IDH1^R132H^): 90-100 nM HT1080 (IDH1^R132C^): ~50 nM BT142 (IDH1^R132H^): ~5 nM	–	–	–	–	–	–	Yes (brain-to-plasma ratio >1 in mouse model)	–	Merck
GSK321 (79)	mutIDH1	Reversible, allosteric, non-competitive inhibition via stabilization of open, inactive enzyme dimer conformation (steric hindrance)	IDH1^R132H^ IDH1^R132C^ IDH1^R132G^	Biochemical: IDH1^R132H^: 4.6 nM IDH1^R132C^: 3.8 nM IDH1^R132G^: 2.9 nM Cell-Based: HT1080 (IDH1^R132C^) (EC_50_): 85nM	–	–	–	–	–	–	–	–	GlaxoSmithKline

### Pan-Inhibitors

#### AG-881

**Figure 2:**
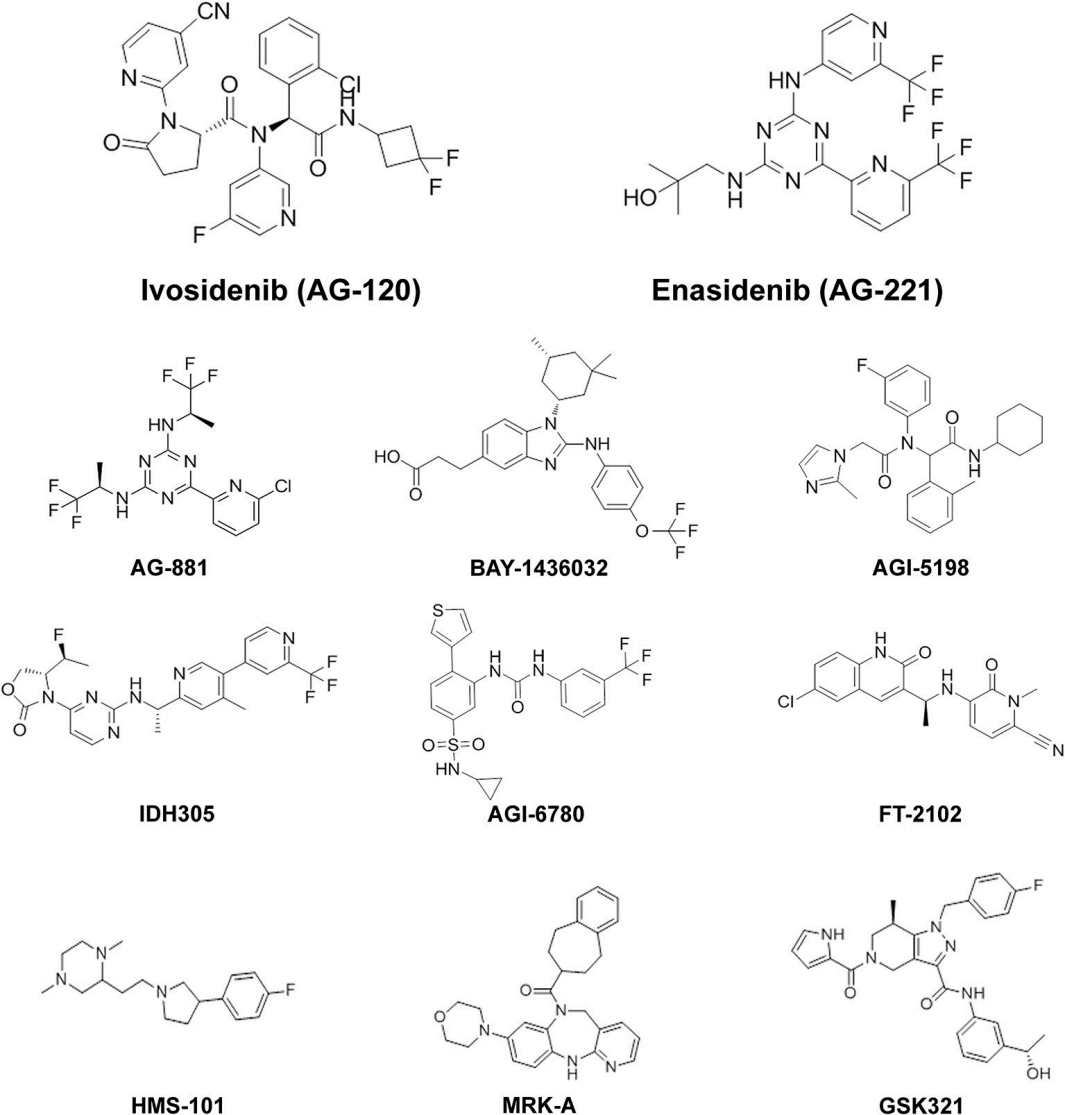
Chemical structures of mutIDH inhibitor compounds reviewed. MutIDH1 inhibitors: Ivosidenib (AG-120), BAY-1436032, AGI-5198, IDH305, FT-2102, HMS-101, MRK-A, GSK321. MutIDH2 inhibitors: Enasidenib (AG-221), AGI-6780. Pan-inhibitors: AG-881.

AG-881 (Vorasidenib) is an orally available pan-inhibitor of both mutIDH1 and mutIDH2 and was the first pan-inhibitor developed under the Celgene and Agios Pharmaceuticals collaboration (Figure 2) (60–62). AG-881 contains a triazine moiety responsible for its allosteric inhibitory activity, and crystallography studies show that AG-881 binds mutIDH1 and mutIDH2 using the same allosteric pocket at the dimer interface, causing steric hindrance that locks the enzymes in an open, inactive conformation (61). Notably, the association of AG-881 with mutIDH1, in particular with IDH1^R132H^, is more efficient than its interaction with mutIDH2 as it achieves maximal potency *in vitro* after significantly shorter incubation periods (61). IC_50_ for inhibition of 2-HG formation following 1 h of preincubation ranged from 6 to 34 nM in both patient-derived and genetically- engineered cell lines expressing IDH1^R132C^, IDH1^R132G^, IDH1^R132H^, IDH1^R132L^, or IDH1^R132S^. For U87 and TF-1 cells transfected with IDH2^R140Q^ or IDH2^R172K^ by lentiviral vector, the IC_50_ values following 1 h of preincubation were 118 nM and 32 nM, respectively (62). In the same study, it was demonstrated that *ex vivo* treatment of primary human AML blasts with AG-881 induced myeloid differentiation (62). AG-881 has also been shown to effectively penetrate the blood-brain barrier in rodents, implicating its potential to treat both IDH-mutant AML and glioma patients (62). Based on this preclinical evidence, two multicenter clinical trials investigating the safety and efficacy of AG-881, one in solid tumors and the other in hematologic malignancies, are currently ongoing (60, 61).

### Specific Inhibitors

#### BAY-1436032

One of the first mutIDH1-specific inhibitors to show preclinical efficacy in both AML and glioma models is BAY-1436032, developed by Bayer. An initial screen of over 3 million compounds based on mutIDH enzymatic activity generated a small group of compounds—with IC_50_ ranging from 0.6 to 17.1 μM—for further evaluation. Optimization of a lead compound based on differential inhibition of mutIDH1 and wild-type IDH1 enzymes resulted in BAY-1436032, an allosteric inhibitor that binds at the IDH dimer interface (Figure 2) (63). Interestingly, BAY-1436032 demonstrates potent inhibition of all known IDH1^R132^ mutants with nearly equal efficacy in both human-derived AML cells (IC_50_ 3–16 nM) and genetically engineered cell lines representative of solid tumors (IC_50_ 13–135 nM) (52, 63). Additionally, reduced proliferation and induction of differentiation was seen *in vitro* in both IDH-mutant AML and glioma cell lines. In AML cell lines, BAY-1436032 demonstrated some efficacy in reducing histone methylation as well, but multiple studies have failed to show changes in histone or DNA methylation status in glioma models (52, 63). *In vivo*, however, BAY-1436032 effectively penetrates the blood-brain barrier and has shown prolonged survival in mice with IDH^R132H^ astrocytoma xenographs (52, 64). Two dose escalation and expansion phase I trials, for AML and solid tumors (including glioma) respectively, are currently ongoing but initial results have yet to be reported (ClinicalTrials.gov NCT03127735, NCT02746081).

#### AGI-5198

A collaboration between Celgene Corporation and Agios Pharmaceuticals for research in cancer metabolism-based therapeutics starting in early 2010 has generated a host of mutIDH inhibitors through a high-throughput screening campaign (65). One of the earliest, and most well-studied mutIDH1-specific inhibitors is AG-5198, a phenyl-glycine-based compound (Figure 2) (66). In early characterization studies, AGI-5198 showed up to 90% 2HG reduction in IDH1^R132H^ U87 xenografts (IC_50_ 0.07 μM *in vitro*) (66, 67). Rohle et al. re-demonstrated the efficacy of AG-5198 in inhibiting 2HG production in patient-derived glioma xenografts and additionally showed that AGI-5198 promotes expression of markers for differentiation, decreases cellular proliferation and decreases histone methylation in the same cell line. However, based on methylation array data, global DNA methylation contributing to the glioma CIMP phenotype was notably unchanged after AGI-5198 treatment (67). A key study by Johannessen et al. using AGI-5198 in an inducible mutIDH1 knock-in human astrocyte model by Johannessen et al. puts these mixed findings into larger context: mutIDH1 inhibition appears to have a small effective time frame, since the role in of mutIDH in gliomagenesis likely changes from “driver” to “passenger.” (80). Early and persistent exposure to AGI-5198 prior to inducing mutIDH1 resulted in reduced 2HG, blocked histone modifications such as methylation, and decreased cellular proliferation; however, just 4 days after the oncogenic insult, the drug was rendered incapable of reversing or blocking the genetic and phenotypic changes rendered by mutIDH (80). Work by Tateishi et al. complements these findings: In multiple patient-derived IDH1^R132H^ glioma tumorsphere lines, treatment with the S-enantiomer of AGI-5198 counterintuitively resulted in modest but consistent increases in cellular proliferation, despite successful depletion of 2HG. Furthermore, it was observed that mice with recurrent mutIDH glioblastoma xenografts had equitable survival and developed similar size tumors between those treated and not treated with the AGI-5198 S-enantiomer (81). The presentation of mixed results in glioma models is likely due to the fact that certain tumorigenic processes are uncoupled from the IDH1 mutation, and further studies into combination treatment (discussed in a later section) may be warranted. Popular hypotheses to explain this changing role of mutIDH include the generation of an epigenetic memory that is indelible by its enzymatic inhibition alone and the accumulation of additional mutations during tumor evolution. The poor pharmacodynamic profile of AGI-5198 due to its rapid metabolism and clearance has precluded its use in clinical trials (68).

#### IDH305

Another mutIDH1-specific inhibitor with demonstrated preclinical efficacy is IDH305, a pyrimidin-5-yl-oxazolidine-2-one compound recently developed by Novartis (Figure 2) (71). IDH305 was developed from efforts to optimize Novartis's first published mutIDH inhibitor, IDH889, as this “parent” drug's high intrinsic clearance, high plasma protein binding, and poor solubility posed significant challenges to further clinical development (82). X-ray crystallography reveals that IDH305 binds to an allosteric binding pocket to stabilize the mutIDH1 enzyme in a catalytically inactive conformation (71). In preclinical characterization testing, IDH305 demonstrated efficacious 2HG reduction in an IDH1^R132H^ colorectal cancer cell line (IC_50_ 24 nM), low liver microsomal clearance values, and substantial brain penetrance in murine models (71). IDH305 has moved into clinical testing in humans with IDH-mutant glioma, AML/MDS, and other solid tumors, and phase 1 safety data in all tumor types is promising (ClinicalTrials.gov NCT02381886) (83).

#### AGI-6780

The first mutIDH2-specific inhibitor to come out of development was AGI-6780, developed as part of the Celgene/Agios collaboration (72). AGI-6780 is a urea sulfonamide inhibitor of the IDH2^R140Q^ mutant enzyme specifically and exhibits non-competitive inhibition with respect to substrate, and uncompetitive inhibition with respect to the NADPH cofactor, operating at an allosteric site at the enzyme's dimer interface (Figure 2) (72, 73). In early pharmacokinetic studies, AGI-6780 demonstrated time-dependent potency for 2HG reduction in both mutant homodimer and mutant/wild-type heterodimer enzyme contexts as well as in more organic cellular contexts (IC_50_ 11nM in IDH2^R140Q^-overexpressing U87 cells) (72). The study of AGI-6780's biological effects is limited to AML-related contexts. The capability for erythropoietin-induced differentiation of TF-1 erythroleukemia cells with IDH2^R140Q^ overexpression is restored after AGI-6780 treatment *in vitro*, and the same effect was seen in patient-derived primary IDH2-mutant AML blood and bone marrow samples cultured *ex vivo* (72). Additional studies have demonstrated that AGI-6780 is not only capable of promoting the expression of differentiation markers such as hemoglobin gamma (HBG) and Kruppel-like factor 1 (KLF1), but that it does so by reversing 2HG-induced DNA and histone hypermethylation in AML cellular models—thereby suggesting that AGI-6780 can manipulate key mechanisms of oncogenesis (72, 74). Lack of *in vivo* evidence and the subsequent development of the broader mutIDH2 inhibitor Enasidenib, discussed below, stunted AGI-6780's further clinical development.

#### Other Isotype-Specific Inhibitors

The remaining group of latest-generation compounds described in the literature include FT-2102, HMS-101, MRK-A, and GSK321—all mutIDH1-specific inhibitors (Figure 2). Despite little preclinical information and an as of yet undisclosed mechanism, FT-2102 is in clinical trials as monotherapy and as combination therapy with azacitidine for AML and MDS, with favorable safety and efficacy data in Phase 1/2 (ClinicalTrials.gov NCT02719574) (75). HMS-101 was an early candidate identified by *in silico* computational screening and validated *in vitro* in murine bone marrow cells transduced with IDH1^R132C^ to deplete 2HG production with an IC_50_ of 1μM and *in vivo* by prolonging survival in a leukemia mouse model (76, 77). MRK-A, developed by Merck, demonstrated effective 2HG reduction in both *in vitro* and *in vivo* glioma models, but yielded mixed results in terms of survival response across various patient-derived glioma xenografts and showed a limited effect on cellular proliferation (78). Lastly, GSK321, developed by GlaxoSmithKline, has been shown to decrease intracellular 2HG in primary IDH1-mutant AML cells *ex vivo* and consequently inhibit cellular proliferation, promote differentiation, and induce global hypomethylation (79). Poor pharmacokinetic properties, especially bioavailability, of GSK321 has limited its clinical use, but modified versions of this compound, such as GSK864, are already undergoing preclinical testing in the developmental pipeline (79, 84).

### FDA-Approved Compounds

#### Enasidenib (AG-221)

The first mutIDH inhibitor given FDA approval was Enasidenib (Idhifa^®^) or AG-221, developed by Celgene Corporation under a license from Agios Pharmaceuticals. Enasidenib is a first-in-class, oral, selective inhibitor of the mutIDH2 enzyme. A precursor form of Enasidenib was originally selected from a high-throughput screen for inhibitors of the most prevalent form of mutIDH2 in AML, IDH2^R140Q^ (47, 51). With triazine substructure-based inhibitory activity, the precursor compound binds mutIDH2 at an allosteric site within the heterodimer interface of the enzyme, much like its predecessor, AGI-6780 (72). X-ray crystallography revealed that this binding at the heterodimer interface forces mutIDH2 to adopt a non-catalytic open conformation, a mechanism of inhibition consistent with conformational functional changes also described in IDH1^R132H^ inhibition (51, 85). Modification of peripheral chemical substituents with additional polar moieties optimized the potency, solubility, clearance and bioavailability profiles of the molecule, yielding Enasidenib (Figure 2) (51).

In initial characterization testing, Enasidenib demonstrated time-dependent potency in reducing 2HG levels in biochemical assays with an IC_50_ of 0.03 μM for the most prevalent oncogenic enzyme, the IDH2^R140Q/WT^ heterodimer, and also for the IDH2^R172K/WT^ enzyme (IC_50_ = 0.01 μM) (51, 86). Robust 2HG depletion was seen in both in transgenic IDH2^R140Q^ TF-1 erythroleukemia cells and in patient-derived primary AML blast cells with either IDH2^R140Q^ or IDH2^R172K^ mutations (51). Furthermore, treatment of patient-derived blasts with Enasidenib also inhibited cellular proliferation and reversed the histone hypermethylation profile associated with the IDH2 mutation (51). *In vivo*, within 10 to 20 days, Enasidenib treatment in mouse models with patient-derived IDH2^R140Q^ AML produced differentiated cells expressing markers such as CD11b, CD14, CD15, and CD24 with a decrease in expression of the immature CD117 marker. Enasidenib treatment also significantly prolonged survival in these models (51). These robust preclinical successes supported further development of the molecule, eventually culminating in demonstration of clinical efficacy in relapsed or refractory AML and FDA approval (11, 12).

#### Ivosidenib (AG-120)

Ivosidenib (Tibsovo^®^), the mutIDH1-specific counterpart to Enasidenib, was developed (also under the Celegene/Agios cancer metabolome research license agreement) out of efforts to optimize AGI-5198 for human therapeutic applications (10). Poor pharmacokinetics of AGI-5198, in particular its prohibitively high metabolic clearance rate, precluded its use in clinical studies despite some success in preclinical testing (66–68). Broad structure-activity relationship profiling directed the substitution of fluorinated cycloalkyl groups in place of cyclohexyl moieties, preventing extensive oxidation of the molecule in the human liver microsome (68). The further addition of a pyrimidine ring restored enzymatic potency after initial modifications, resulting in a metabolically-optimized prototype, AG-14100 (68). However, assessment in early screening however revealed that AG-14100 was a relatively potent cytochrome P450 (CYP) 3A4-inducer, with approximately 70% of the activity of rifampicin. Further functional group substitutions to increase polarity resulted in a molecule with low CYP 3A4 activation, and adequate clearance and potency profiles: AG-120 (Ivosidenib) (Figure 2) (68).

Ivosidenib is a highly specific, allosteric, reversible inhibitor of mutIDH1. It competes for binding with the enzyme's essential cofactor, the magnesium ion, thereby preventing the formation of a catalytically active site (87). It possesses equitable potency across various IDH1^R132^ mutants in a series of cell lines in a selective manner, showing no inhibition of wild-type or mutIDH2 isoforms (68). Testing in animal models with an intact blood-brain barrier suggests a modest level of brain penetration (4.1%), which is hypothesized to be increased in glioma patients with some intrinsic blood-brain barrier disruption (68). Robust, dose- and time-dependent 2HG depletion has been observed across a host of cell types including human chondrosarcoma cells and mouse-model xenografts, primary human AML myeloblasts, and mutIDH1^R132H^ glioma xenografts (IC_50_ range 5–13 nM for various IDH1 mutants *in vitro*) (68, 88, 89). Furthermore, preclinical evidence that Ivosidenib modulates oncogenic properties of cancer cells (in addition to 2HG reduction) has been repeatedly demonstrated by induction of cellular differentiation in AML myeloblasts and through inhibition of cellular migration and invasion in a chondrosarcoma cell line (88–90). These early preclinical successes, followed by phase I clinical trials demonstrating a favorable safety profile, earned Ivosidenib orphan drug status for glioma in January 2018, and its first global approval by the FDA in July 2018 for adults with refractory or relapsed AML (10).

## Clinical Trials

### Clinical Trials in AML

A summary of the completed, ongoing, recruiting, and planned clinical trials of mutIDH inhibitors in AML and other hematological malignancies can be found in Table 2 and will be reviewed below.

**Table 2:** Clinical trials in AML/hematologic malignancies.

**Compound**	**NCT No**	**Title**	**PHASE**	**MODEL**	**STATUS**	**indication(S)**	**intervention ARM(s)**	**enrollment**	**SPONSOR(S)**
ENASIDENIB (AG-221)	NCT01915498	A phase 1/2, Multicenter, Open-Label, Dose-escalation and expansion, Safety, Pharmacokinetic, Pharmacodynamic, and clinical activity study of orally administered AG-221 in subjects with advanced hematologic malignancies with an IDH2 mutation	1/2	Single group assignment, open label	Active, not recruiting	AML, MDS with IDH2 mutation	AG-221 administered orally daily during 28-days cycles until disease progression or toxicity; Multiple doses	357	Celgene, Agios
	NCT02273739	A phase 1/2, Multicenter, Open-Label, Dose-escalation study of AG-221 in subjects with advanced solid tumors, Including glioma, and With Angioimmunoblastic T-cell lymphoma, That harbor an IDH2 mutation	1/2	Single group assignment, open label	Completed	Solid Tumor, Glioma, Angioimmunoblastic T-cell Lymphoma, Intrahepatic Cholangiocarcinoma, Chondrosarcoma with IDH2 mutation	AG-221 administered orally daily during 28-days cycles until disease progression or toxicity; Multiple doses	21	Celgene
	NCT03515512	A phase I study of IDH2 inhibition using enasidenib as maintenance therapy for IDH2-mutant myeloid neoplasms following allogeneic stem cell Transplantation	1	Single group assignment, open label	Recruiting	AML, MDS, CMML with IDH2 mutation undergoing first hematopoietic stem cell transplantation	AG-221 administered orally daily during 28-days cycles	22 (est)	Massachusetts general hospital, Celgene
	NCT03683433	Phase II study of the targeted mutant IDH2 inhibitor enasidenib in combination with azacitidine for relapsed/refractory AML	2	Single group assignment, open label	Recruiting	AML, MDS, Biphenotypic/bilineage leukemia who have failed prior therapy with IDH2 mutation	Subcutaneous or intravenous azacitidine on days 1–7, and Enasidenib daily starting day 1 with repeated courses every 4–6 weeks in absence of disease progression or toxicity	50 (est)	MD Anderson, NCI
	NCT02577406	A phase 3, Multicenter, Open-label, Randomized study comparing the efficacy and safety of AG-221 (CC-90007) Vs. Conventional care regimens in older subjects with late stage acute myeloid leukemia harboring an isocitrate dehydrogenase 2 mutation (IDHENTIFY)	3	Randomized, parallel assignment, open label	Recruiting	Age 60+, primary or secondary relapsed/refractory AML with IDH2 mutation	1. AG-221 and best supportive care 2. Best supportive care 3. Subcutaneous azacitidine and best supportive care 4. Intermediate-dose cytarabine and best supportive care	316 (est)	Celgene
	NCT03728335	Phase IB trial of enasidenib (AG-221) maintenance post allogeneic hematopoietic cell transplantation in patients with IDH2 mutation	1	Single group assignment, open label	Not yet recruiting	AML with IDH2 mutation and has received a hematopoietic stem cell transplantation	AG-221 administered orally daily during 28-days cycles for up to 24 courses	15 (est)	City of hope medical center, NCI
	NCT03720366	A phase 1, 2-Part, Multicenter, Open-label, 3-Arm study to determine the effect of enasidenib (CC-90007) on the pharmacokinetics of single doses of caffeine, Dextromethorphan, Flurbiprofen, Midazolam, Omeprazole, Digoxin, Rosuvastatin, and pioglitazone in subjects with acute myeloid leukemia harboring an isocitrate dehydrogenase 2 mutation	1	Non-randomized, parallel assignment, open label	Not yet recruiting	Primary or secondary relapsed/refractory AML with IDH2 mutation	1. AG-221 with “probes”: caffeine, dextromethorphan, flurbiprofen, midazolam and omeprazole 2. AG-221 with “probes”: digoxin and rosuvastatin 3. AG-221 with “probes”: pioglitazone	42 (est)	Celgene
IVOSIDENIB (AG-120)	NCT02074839	A phase I, multicenter, Open-Label, Dose-escalation and expansion, Safety, Pharmacokinetic, Pharmacodynamic, and Clinical activity study of orally administered AG-120 in subjects with advanced hematologic malignancies with an IDH1 mutation	1	Single group assignment, open label	Active, not recruiting	Advanced hematologic malignancy with IDH1 mutation	AG-120 administered orally daily during 28-day cycles until disease progression, toxicity, or hematopoietic stem cell transplant	266	Agios
	NCT03245424	Expanded access program for ivosidenib (AG-120) monotherapy in patients with relapsed or refractory acute myeloid leukemia with an IDH1 mutation		Expanded access	Approved for marketing	Relapsed/refractory AML with IDH1 mutation	AG-120 500 mg administered orally daily during 28-days cycles		Agios
	NCT03471260	Phase Ib/II investigator sponsored study of the IDH1-mutant inhibitor ivosidenib (AG120) with the BCL2 inhibitor venetoclax in IDH1-mutated hematologic malignancies	1b/2	Single group assignment, open label	Recruiting	Relapsed/refractory AML, MDS, MPN with IDH1 mutation	Venetoclax orally daily on days 1–14 of each 28-days cycle, AG-120 orally daily starting on day 15 of cycle 1	48 (est)	MD Anderson, AbbVie, Agios
	NCT03173248	A Phase 3, Multicenter, Double-Blind, Randomized, Placebo-controlled study of AG-120 in combination with azacitidine in subjects ≥ 18 Years of age with previously untreated acute myeloid leukemia with an IDH1 mutation	3	Randomized, parallel assignment, triple blind	Recruiting	Untreated AML with IDH1 mutation	1. AG-120 with azacitidine 2. Placebo with azacitidine	392 (est)	Agios
	NCT03564821	A phase 1 study of IDH1 inhibition using ivosidenib as maintenance therapy for IDH1-mutant myeloid neoplasms following allogeneic stem cell transplantation	1	Single group assignment, open label	Not yet recruiting	AML, MDS, CMML with IDH1 mutation undergoing hematopoietic stem cell transplantation	AG-120 500 mg or 250 mg administered orally daily during 28-days cycles	22 (est)	Massachusetts general hospital, Agios
	NCT03503409	A single-arm phase II multicenter study of IDH1 (AG 120) inhibitor in patients with IDH1 mutated myelodysplastic syndrome	2	Single group assignment, open label	Not yet recruiting	MDS and non-proliferative AML (up to 29% blasts) with IDH1 mutation	AG-120 500 mg administered orally daily during 28-days cycles	68 (est)	Groupe francophone des myelodysplasies
Ivosidenib (AG-120) + Enasidenib (AG-221)	NCT02632708	A phase 1, Multicenter, Open-label, Safety study of AG-120 or AG-221 in combination with induction therapy and consolidation therapy in patients with newly diagnosed acute myeloid leukemia with an IDH1 and/or IDH2 mutation	1	Non-randomized, parallel assignment, open label	Active, not recruiting	Newly diagnosed or untreated AML, AML arising from MDS, AHD, or after exposure to genotoxic injury with IDH1 or IDH2 mutation	1. AG-120 with cytarabine and daunorubicin 2. AG-120 with cytarabine and idarubicin 3. AG-221 with cytarabine and daunorubicin 4. AG-221 (starting on day 8) with cytarabine and idarubicin 5. AG-221 (starting on day 8) with cytarabine and daunorubicin 6. AG-221 with cytarabine and idarubicin	153	Agios, Celgene
	NCT02677922	A phase 1b/2 Open-label, Randomized study of 2 combinations of isocitrate dehydrogenase (IDH) mutant targeted therapies plus azacitidine: oral AG-120 plus subcutaneous azacitidine and oral AG-221 plus SC azacitidine in subjects with newly diagnosed acute myeloid leukemia harboring an IDH1 or an IDH2 mutation, respectively, who are not candidates to receive intensive induction chemotherapy	1b/2	Randomized, parallel assignment, open label	Active, not recruiting	Primary or secondary AML with an IDH1 or IDH2 mutation	1. AG-120 with subcutaneous azacitidine 2. AG-221 with subcutaneous azacitidine 3. Subcutaneous azacitidine	131	Celgene
	NCT03013998	A master protocol for biomarker-based treatment of AML (The beat AML trial)	1/2	Non-randomized, parallel assignment, open label	Recruiting	Group A: Age 60+, previously untreated AML Group B: Age 60+, relapsed/refractory AML	Arm assignment made based on initial molecular biomarker analysis: 1. Samalizumab, daunorubicin, cytarabine 2. BI 836858, azacitidine 3. AG-221, azacitidine 4. Entospletinib, azacitidine 5. Entospletinib, decitabine 6. Entospletinib, azacitidine, daunorubicin, cytarabine 7. Pevonedistat, azacitidine 8. AG-120, azacitidine 9. Gilteritinib, decitabine	500 (est)	Beat AML, LLC
AG-881	NCT02492737	A phase I, Multicenter, Open-Label, Dose-escalation and expansion, Safety, Pharmacokinetic, Pharmacodynamic, and clinical activity study of orally administered AG-881 in patients with advanced hematologic malignancies with an IDH1 and/or IDH2 mutation	1	Single group assignment, open label	Completed	Advanced hematologic malignancy with IDH1 or IDH2 mutation	AG-881 administered orally daily during 28-days cycles until disease progression or toxicity	46	Agios
BAY-1436032	NCT03127735	An open-label, Non-randomized, Multicenter phase I study to determine the maximum tolerated and / or recommended phase II dose of oral mutant IDH1 (mIDH1) inhibitor BAY1436032 and to characterize its safety, Tolerability, Pharmacokinetics, Pharmacodynamics, and preliminary clinical efficacy in patients with mIDH1-R132X advanced acute myeloid leukemia (AML)	1	Single group assignment, open label	Active, not recruiting	Advanced relapsed/refractory AML with IDH1 mutation	BAY-1436032 administered orally twice daily for 28-days cycles until disease progression or toxicity	27	Bayer
IDH305	NCT02381886	A phase I study of IDH305 in patients with advanced malignancies that harbor IDH1R132 mutations	1	Single group assignment, open label	Active, not recruiting	Advanced malignancy with IDH1 mutation	IDH305	166	Novartis
FT-2102	NCT02719574	A phase 1/2, Multicenter, Open-label study of FT-2102 as a single agent and in combination with azacitidine or cytarabine in patients with acute myeloid leukemia or myelodysplastic syndrome with an IDH1 mutation	1/2	Non-randomized, parallel assignment, open label	Recruiting	Relapsed/refractory AML, MDS with IDH1 mutation	Dose Escalation and Expansion: 1. FT-2102 at 50 mg or 150 mg 2. FT-2102 at 50 mg or 150 mg with azacitidine 3. FT-2102 at 50 mg or 150 mg with cytarabine Experimental: 4. FT-2102 at 50 mg or 150 mg for relapsed/refractory AML 5. FT-2102 at 50 mg or 150 mg for AML/MDS in complete remission 6. FT-2102 at 50 mg or 150 mg for relapsed/refractory AML previously treated with mutIDH1 inhibitor 7. FT-2102 at 50 mg or 150 mg with azacitidine for hypomethylating agent-naïve patients 8. FT-2102 at 50 mg or 150 mg with azacitidine for patients who previously inadequately responded to hypomethylating agents 9. FT-2102 at 50 mg or 150 mg with azacitidine for patients who have previously been treated with mutIDH1 inhibitor	400 (est)	Forma

### MutIDH2 Inhibitors

The safety and efficacy of Enasidenib demonstrated in a phase 1/2 trial in IDH2-mutant advanced myeloid malignancies (including AML and MDS) paved the way for FDA approval of the first small molecule mutIDH inhibitor in cancer and propelled global clinical research efforts for IDH-based targeted cancer treatments (ClinicalTrials.gov NCT01915498) (12). In this landmark first-in-human dose-escalation and expansion study, the pharmacokinetic and pharmacodynamic profiles of Enasidenib were first established along with evidence for clinical efficacy in patients with relapsed/refractory AML. In the initial dose-escalation part of the study, 176 patients were administered doses of Enasidenib ranging from 50 to 650 mg daily in 28-days cycles and a maximum tolerated dose (MTD) was not reached. A 100 mg daily dose was chosen for the 109 patients in the dose expansion arm based on a maximized plasma depletion of 2HG of up to 99% in IDH2^R140Q^ patients and up to 94% in IDH2^R172K^ patients. In patients with relapsed or refractory AML, 38.5% of patients receiving the expansion dose demonstrated a clinical response with a median 5.6 months response duration (8.8 months in those who initially achieved a complete response) (12). Unlike in standard cytoreductive chemotherapy, treatment response occurred without a period of bone marrow hypoplasia, and bone marrow aspirates revealed that responders were characterized by robust myeloid differentiation and trilineage hematopoietic recovery. The most common treatment-related adverse events were indirect hyperbilirubinemia (38%) and nausea (23%), and IDH-inhibitor-associated differentiation syndrome (IDH-DS) was one of the most frequent high-grade adverse events (6%) (12). A similarly designed trial of Enasidenib in IDH2-mutant solid tumors, including glioma, was completed in 2016, but data from the trial has not yet been published (ClinicalTrials.gov NCT02273739).

Since the success of the Enasidenib phase 1/2 trial in myeloid malignancies and FDA approval for the indication of relapsed/refractory IDH2-mutant AML was obtained in 2017, clinical trials investigating the efficacy of mutIDH2 inhibitors, mostly Enasidenib, in various AML subpopulations have become abundant. One, which is actively recruiting, is analyzing the safety of Enasidenib when given in combination with standard AML induction and consolidation chemotherapy in newly diagnosed AML (ClinicalTrials.gov NCT02632708). Another recruiting phase 1 trial is investigating Enasidenib as a maintenance therapy for IDH2-mutant AML or CML following allogeneic hematopoietic stem cell transplantation (ClinicalTrials.gov NCT03515512). Two trials, a phase 1b/2 and a phase 2, are evaluating Enasidenib in combination with a DNA methyltransferase (DNMT) inhibitor, azacitidine, in newly-diagnosed and relapsed/refractory AML, respectively (ClinicalTrials.gov NCT02677922, NCT03683433). Another phase 1b/2 trial is studying biomarker-based treatment of AML more broadly by using genomic screening to identify specific subtypes of AML (including IDH2-mutant) and assign each subtype to a distinct treatment regimen—IDH2-mutant AML will be assigned to Enasidenib treatment (ClinicalTrials.gov NCT03013998). There is one phase 3 randomized, open-label study comparing Enasidenib to conventional care specifically in patients age 60 or older with relapsed/refractory IDH2-mutant advanced AML, also known as IDHENTIFY (ClinicalTrials.gov NCT02577406). The IDHENTIFY protocol was presented at the 2017 American Society of Clinical Oncology (ASCO) Annual Meeting (91). A few clinical trials involving Enasidenib are still in the planning stages and are not yet actively recruiting (ClinicalTrials.gov NCT03728335, NCT03720366), and one phase 1 trial investigating pan-inhibitor AG-881 (ClinicalTrials.gov NCT02492737) in advanced hematologic malignancies with either IDH1 or IDH2 mutations concluded in 2018, but no trial results have been released.

#### MutIDH1 Inhibitors

Shortly after the approval of Enasidenib, its mutIDH1 counterpart, Ivosidenib, similarly demonstrated safety and clinical efficacy in a phase 1/2 dose-escalation/dose-expansion trial of relapsed/refractory IDH1-mutant AML (ClinicalTrials.gov NCT02074839) (13). In this study, the primary efficacy group included 125 patients with relapsed/refractory IDH1-mutant AML who received 500 mg of Ivosidenib daily for at least 6 months. The 500 mg daily dose was chosen during the dose-escalation phase as it was noted that no additional inhibition of 2HG in plasma was observed at higher doses, and at this dose, a decrease in plasma 2HG to the level of healthy persons was achieved in most patients. In the primary efficacy group, complete remission or complete remission with partial hematologic recovery was seen in 30.4% of patients and the overall response rate was 41.6%. The median duration of overall response was 6.5 months and overall survival was 8.8 months. Interestingly, of the 30.4% of patients with complete remission, 21% had no detectible residual IDH1 mutations on digital polymerase chain reaction assay. These patients had significantly longer durations of remission (11.1 vs. 6.5 months) and overall survival (14.5 vs. 10.2 months) than those who did not clear the mutation (13). However, no preexisting gene mutation or other genetic status significantly predicted treatment success or failure, although increased mutations in receptor tyrosine kinase pathway genes were more strongly associated with non-responders. As in the Enasidenib trials, induction of myeloblast differentiation, as seen on bone marrow aspirates, seemed to drive the clinical efficacy of Ivosidenib. Most frequently observed high-grade treatment-related adverse events included QT prolongation (7.8%) and IDH-DS (3.9%) (13). Shortly after the publication of these results, the FDA approved its second mutIDH inhibitor, Ivosidenib, for IDH1-mutant relapsed or refractory AML (10). Additionally, Ivosidenib now also has an expanded access program for this patient population (ClinicalTrials.gov NCT03245424).

There are several trials studying Ivosidenib in AML in various treatment contexts. The previously mentioned currently recruiting phase 1b/2 study investigating biomarker-based treatment of AML also includes an Ivosidenib arm for those patients with IDH1 mutations (ClinicalTrials.gov NCT03013998). Another trial in the recruitment phase is examining simultaneous treatment with Ivosidenib and the chronic lymphocytic leukemia drug venetoclax in patients with relapsed or refractory AML (NCT03471260). In another currently-recruiting phase 3 study azacitidine is combined with either Ivosidenib or placebo as therapy for previously untreated AML with IDH1 mutations (NCT03173248). This study is planning on enrolling 392 patients, but data as of September 2017 indicated that out of 11 patients who received both Ivosidenib and azacitidine, 8 of them achieved a response, including 4 who had complete remission (92). As mentioned above, another active phase 1 study is examining treatment with either Ivosidenib or Enasidenib, depending on IDH1 vs. IDH2 mutational status, alongside standard induction or consolidation therapy for AML (ClinicalTrials.gov NCT02632708). In preliminary results looking at patients treated with Ivosidenib, 12 of 14 patients with primary AML and 4 of 9 patients with secondary AML achieved either complete remission, complete remission with incomplete platelet recovery, or complete remission with incomplete hematologic recovery (93). Preliminary results of another phase 1b/2 study looking at Ivosidenib with subcutaneous azacitidine in newly diagnosed AML were presented at the 2018 ASCO Annual Meeting; of 23 patients taking both Ivosidenib and azacitidine, 18 (78%) received a response, of whom 10 had complete remission and the median time to response was 1.8 months (ClinicalTrials.gov NCT02677922) (94). There are also a handful of additional trials of Ivosidenib for hematological malignancies that are still in the planning stages (ClinicalTrials.gov NCT03564821, NCT03503409).

Clinical trials experimenting with other mutIDH1 inhibitors in hematologic malignancy have also taken off. An open-label phase 1 trial of BAY-1436032 in patients with IDH1^R132^-mutant AML started in 2017 with no results published to date (ClinicalTrials.gov NCT03127735). A phase 1/2 study is evaluating the use of FT-2102 alone or with azacitidine in patients with AML or MDS carrying IDH1 mutations (ClinicalTrials.gov NCT02719574). Results presented at the 2018 ASCO Annual Meeting from data on 57 patients (31 treated with FT-2102, 27 treated with both FT-2102 and azacitidine) demonstrated a complete response rate of 38% with monotherapy and 27% with combination therapy (75). Lastly, there is an ongoing phase 1 study looking at IDH305 in patients with diverse IDH1 mutation-harboring malignancies, including glioma, AML/MDS, and other solid tumors (ClinicalTrials.gov NCT02381886). In the AML/MDS cohort (*n* = 24), complete remission was achieved in 2 patients (9.5%), complete remission with incomplete recovery in 1 patient (4.8%), and partial remission in 4 patients (19%) (83). Notably, early adverse event data from this study has halted clinical development of IDH305 because of dose-limiting toxicities such as hyperbilirubinemia and transaminitis (64).

### Clinical Trials in Glioma and Other Solid Tumors

Several clinical trials studying mutIDH inhibitors in glioma and other solid tumors are ongoing or in planning stages and involve both pan and specific inhibitors (Table 3). Early results from ongoing phase 1 safety and dose-escalation trials continue to be updated annually during national conferences (69, 95–100).

**Table 3:** Clinical trials in glioma and other solid tumors.

**Compound**	**NCT No**	**Title**	**PHASE**	**MODEL**	**STATUS**	**Indication(S)**	**Intervention ARM(s)**	**Enrollment**	**SPONSOR(S)**
IVOSIDENIB(AG-120)	NCT02073994	A phase 1, Multicenter, Open-label, Dose-escalation and expansion, Safety, Pharmacokinetic, Pharmacodynamic, and clinical activity study of orally administered AG-120 in subjects with advanced solid tumors, including glioma, With an IDH1 mutation	1	Single group assignment, open label	Active, not recruiting	Cholangiocarcinoma, chondrosarcoma, non-enhancing glioma, other solid tumors with IDH1 mutation	AG-120 administered orally daily during 28-days cycles until disease progression or toxicity	170 (est)	Agios
	NCT02989857	A phase 3, Multicenter, Randomized, Double-blind, Placebo-controlled study of AG-120 in previously-treated subjects with nonresectable or metastatic cholangiocarcinoma with an IDH1 mutation	3	Randomized, parallel assignment, double blind	Recruiting	Advanced or metastatic cholangiocarcinoma with IDH1 mutation	1. AG-120 500 mg daily continuous dosing 2. Matched placebo; those that experience disease progression will be allowed to cross-over	186 (est)	Agios
AG-881	NCT02481154	A phase 1, Multicenter, Open-label, Dose-escalation and expansion, Safety, Pharmacokinetic, Pharmacodynamic, and Clinical activity study of orally administered AG-881 in patients with advanced solid tumors, Including Gliomas, With an IDH1 and/or IDH2 mutation	1	Single group assignment, open label	Active, not recruiting	Glioma with IDH1 or IDH2 mutation	AG-881 administered orally daily during 28-days cycles until disease progression or toxicity	150 (est)	Agios
Ivosidenib (AG-120) + AG-881	NCT03343197	A phase 1, Multicenter, Randomized, Controlled, Open-label, Perioperative study of AG-120 and AG-881 in subjects with recurrent, Non-enhancing, IDH1 mutant, Low grade glioma	1	Randomized, parallel assignment, open label	Recruiting	WHO grade II or III low-grade glioma with IDH1 mutation and primarily non-enhancing disease	1. No pre-surgical treatment 2. AG-881 administered daily for 1 28-days cycle prior to surgery with the option to continue afterwards 3. AG-120 administered daily for 1 28-days cycle prior to surgery with the option to continue afterwards	45 (est)	Agios
DS-1001b	NCT03030066	A phase 1 study of DS-1001b in patients with IDH1 mutated gliomas	1	Single group assignment, open label	Recruiting	Glioma with IDH1 mutation	DS-1001b	60 (est)	Daiichi Sankyo Co.
IDH305	NCT02381886	A phase I study of IDH305 in patients with advanced malignancies that harbor IDH1R132 mutations	1	Single group assignment, open label	Active, not recruiting	Advanced malignances with IDH1 mutation	IDH305	166	Novartis
FT-2102	NCT03684811	A Phase 1b/2 Study of FT 2102 in participants with advanced solid tumors and gliomas with an IDH1 mutation	1b/2	Non-randomized, parallel assignment, open label	Recruiting	Recurred/progressed glioma, hepatobiliary cancer, chondrosarcoma, intrahepatic cholangiocarcinoma (non-resectable) with IDH1 mutation	1. FT-2102 150 mg per protocol (phase 1b) 2. FT-2102 150 mg per protocol (phase 2) 3. FT-2102 150 mg per protocol with azacitidine (phase 1b+2 combined) 4. FT-2102 150 mg per protocol with nivolumab (phase 1b+2 combined) 5. FT-2102 150 mg per protocol with gemcitabine and cisplatin (phase 1b+2 combined)	200 (est)	Forma
ENASIDENIB(AG-221)	NCT02273739	A phase 1/2, Multicenter, Open-Label, Dose-escalation study of AG-221 in subjects with advanced solid tumors, including glioma, and with angioimmunoblastic T-cell lymphoma, that harbor an IDH2 mutation	1/2	Single group assignment, open label	Completed	Recurred/progressed Advanced solid tumor (including glioma, angioimmunoblastic T-cell lymphoma)	AG-221 administered orally daily during 28-days cycles until disease progression or toxicity	21	Celgene
BAY-1436032	NCT02746081	An Open-label, Non-randomized, Multicenter phase I study to determine the maximum tolerated or recommended phase II dose of oral mutant IDH1 inhibitor BAY1436032 and to characterize its safety, Tolerability, Pharmacokinetics and preliminary pharmacodynamic and anti-tumor activity in patients with IDH1-R132X-mutant advanced solid tumors	1	Single group assignment, open label	Active, not recruiting	Solid tumors with IDH1 mutation	BAY-1436032 300 mg administered orally daily in 21-days cycles	81	Bayer

In 2015, the first phase 1 data on the safety of Ivosidenib in patients with advanced glioma and other IDH-mutant solid tumors was presented at the annual EORTC-NCI-AAR Molecular Targets and Cancer Therapeutics Symposium and indicated that Ivosidenib treatment was well-tolerated with a positive pharmacokinetic profile (ClinicalTrials.gov NCT02073994) (95). This multicenter, open-label study was designed as a dose-escalation study with a following dose-expansion cohort. Ivosidenib was administered daily over 28-days cycles at doses ranging from 100 mg up to 1200 mg. Doses beyond 500 mg did not result in increased plasma 2HG reduction; therefore the 500 mg dose was selected for the dose-expansion arms, which included both enhancing and non-enhancing IDH-mutant gliomas (97). Most recent efficacy data reported at the 2016 Society for Neuro-Oncology Annual Meeting indicated a 35% clinical benefit rate (stable disease or better) based on imaging at 6-months follow-up (96). By 2017, 168 patients were enrolled in the dose-escalation arm and 108 patients in the 500 mg daily dose-expansion arm (97). Throughout the study period, Ivosidenib has continued to demonstrate good oral bioavailability, a lengthy half-life (mean 40–102 h after a single dose), and a persistent, robust response in 2HG depletion in both plasma and tumor tissue (97). No treatment-related serious adverse events have been reported; other adverse events included headache, nausea, fatigue, and gastrointestinal symptoms (96).

AG-881 (Vorasidenib), a pan-inhibitor, is the only other mutIDH inhibitor currently with supporting clinical data in glioma. In a similarly designed, phase 1, multicenter, open-label, dose-escalation and expansion trial, the safety and pharmacokinetic profiles of AG-881 is being investigated in both mutIDH1 and mutIDH2 gliomas and other solid tumors (ClinicalTrials.gov NCT02481154) (69, 98). According to the most recent data presented at the 2018 Society for Neuro-Oncology Annual Meeting, 52 glioma patients have received treatment with AG-881 on a 28-days cycle either as part of the dose-escalation arm (dose range 25–300 mg daily) or as part of the dose-expansion arm (10 or 50 mg daily) (69). While preliminary efficacy data has yet to be published, the most frequently observed adverse events included elevation of transaminases (ALT 44.2 and AST 38.5%) and headache (34.6%). Furthermore, at doses >100 mg, five subjects experienced dose-limiting toxicity presenting as liver injury (69). Forthcoming work with AG-881 includes an additional ongoing phase 1 randomized open-label trial evaluating the ability of pre-treatment with either AG-881 or AG-120 to suppress intra-tumoral 2HG levels in surgical pathology specimens as a measure of pharmacological efficacy (ClinicalTrials.gov NCT03343197). This trial will use the 10 or 50 mg daily dosing (69, 98, 101). The protocol for the latter study was likewise recently presented at the 2018 Society for Neuro-Oncology Annual Meeting (101).

Other ongoing clinical trials of mutIDH inhibitor compounds in glioma (currently without early results) are also in phase 1 and are mostly in the early recruitment phase. These studies are evaluating safety and pharmacology of mutIDH-specific inhibitors such as DS-1001b, IDH305, and FT-2012, Enasidenib, and BAY-1436032 (ClinicalTrials.gov NCT03030066, NCT02381886, NCT03684811, NCT02273739, NCT02746081). There is a single phase 3, multicenter, randomized, double-blind study in solid tumors comparing Ivosidenib (500 mg daily) to placebo in advanced or metastatic mutIDH1 cholangiocarcinoma, also known as the “ClarIDHy” trial (ClinicalTrials.gov NCT02989857) (100). The “ClarIDHy” protocol was presented at the 2017 ASCO Annual Meeting based on previous phase 1 trial findings of 6% partial improvement and 56% stable tumor response, and a progression-free survival rate of 40% at 6 months in a similar patient population (ClinicalTrials.gov NCT02073994) (100).

## Adverse Events and the IDH-Inhibitor-Associated “Differentiation Syndrome”

It should be noted that mutIDH inhibitors are not without significant side effects to which patients have differential susceptibilities depending on whether they are being treated for a solid or a hematologic malignancy. Reported adverse events in trials for solid tumors are fewer and far more manageable: In the phase 1 studies for AG-881, the most common adverse events included fatigue (30.8–38.7%), nausea (26.9–35.5%), and dose-dependent transaminase elevation (without elevated bilirubin) (34.4–44.2%) (69, 98). All dose-related toxicities were reversible with dose modification to the eventual expansion arm dose of 50 mg or with a short period of discontinuation (98). Likewise, in the Ivosidenib studies in both glioma and cholangiocarcinoma, the most frequently occurring adverse events included fatigue (41%), nausea (21.8–36%), and diarrhea (16.4–30%) (95, 102). Only one patient in the dose-escalation arm of the cholangiocarcinoma cohort required a dose reduction to the expansion arm dose (1,200 mg reduced to 500 mg) for suspected drug-related muscle cramps (102).

Patients with hematologic malignancy, on the other hand, experience a greater incidence of more critical treatment-related adverse events that are more critical in nature. As previously discussed, 2017 and 2018 saw the respective publications of the results of two landmark clinical trials in AML: first, a phase 1/2 study of the selective IDH2 mutant inhibitor Enasidenib, and second, a phase 1 dose-escalation and dose-expansion study of the selective IDH1 mutant inhibitor Ivosidenib (12, 13). In the Enasidenib trial, approximately four of every five patients had a treatment-related adverse event, the most common overall being indirect hyperbilirubinemia (38%) and nausea (23%). Indirect hyperbilirubinemia was presumed to be the result of a benign, Gilbert syndrome—like mechanism, as no evidence was found in these patients of Enasidenib-induced hepatotoxicity. Additionally, as previously discussed, the IDH-inhibitor—associated differentiation syndrome (IDH-DS) was also one of the most frequent high-grade treatment-related adverse events (6%) (12). Similarly, in the Ivosidenib trial, approximately one in five patients experienced at least one severe adverse event, the most common being QT interval prolongation (7.8%) and IDH-DS (3.9%). Notably, no patients were required to permanently discontinue Ivosidenib as a result of these adverse events and there were no Ivosidenib-related fatalities, but two patients in the phase 1 Enasidenib trial died due to complications of IDH-DS (12, 13). IDH-DS has been reported as a severe adverse effect of interest in AML trials across nearly all of the mutIDH inhibitors, and warrants further discussion (12, 13, 64, 75).

Differentiation syndrome (DS), previously called “retinoic acid syndrome,” was originally described over a quarter-century ago as an adverse event in patients with acute promyelocytic leukemia (APL) undergoing treatment with all-*trans* retinoic acid (ATRA) (103). This life-threatening syndrome is typically seen within 3 weeks of ATRA treatment initiation and consists primarily of fever and respiratory distress, with other notable findings including weight gain, lower extremity edema, pleural or pericardial effusions, and episodic hypotension. In the originally described cohort, three patients died from complications of “retinoic acid syndrome,” while high-dose dexamethasone administered early in the course of the syndrome was found to be efficacious in treating those patients who survived (103). A similar differentiation syndrome has also been described following arsenic trioxide treatment in APL (104).

IDH-DS can first develop up to several months following the initiation of IDH mutant inhibitor treatment for hematologic malignancy and shares distinctive symptomology with the retinoic acid syndrome: culture-negative fever, rapid weight gain or edema, respiratory symptoms with or without infiltrates, pleural or pericardial effusions, hypotension, and acute renal failure (105, 106). Diagnosis is often challenging because symptomology is also similar to that of leukemic progression (105, 107–109). Shortly after the 2017 phase 1 Enasidenib trial in AML, an independent differentiation syndrome review committee (DSRC) retrospectively analyzed possible cases of IDH-DS, identified distinguishing characteristics of patients who developed IDH-DS, such as fewer previous anticancer therapies and higher baseline peripheral blasts and lactate dehydrogenase levels, and outlined an easy-to-follow protocol for IDH-DS diagnosis and management (105). Similar to treatment for DS in APL, treatment for IDH-DS is dexamethasone 10 mg twice daily until IDH-DS symptoms have significantly improved. If associated with severe pulmonary or renal symptoms, mutIDH inhibitor therapy should be halted until IDH-DS symptom resolution (105). Table 4 summarizes current knowledge and consensus concerning diagnosis and management of DS more generally (103, 105, 106, 110–115).

**Table 4:** Differentiation syndrome: clinical features and management.

Predictive factors	• Body mass index ≥ 30 (kg/m^2^) (110, 111). • High peripheral blast cell % on admission (110, 112). • White blood cell (WBC) count greater than 10x10^9^/L on admission (112). • Abnormally high lactate dehydrogenase (LDH) level on admission (112). • Bone marrow blast count ≥ 20% on admission (105). • Previously undergone fewer antileukemic regimens (105). • Female sex (113).
Clinical features (103, 105, 112)	• General: unexplained fever (>38°C), peripheral edema/weight gain of >5 kg, hypotension, tachycardia, lymphadenopathy, arthralgias, rash • Cardiopulmonary: dyspnea, hypoxia, pleural/pericardial effusion, pericarditis, pulmonary infiltrates • Renal: acute kidney injury • Hematological: leukocytosis (with mature neutrophil predominance and decreased blast count), disseminated intravascular coagulation (DIC)
Diagnostics (112, 114)	There are no laboratory values or imaging findings indicative of DS, but features to monitor for severity include: • Imaging: • Chest X-ray: may find cardiomegaly, septal lines, pleural effusion, patchy infiltrates • Chest CT: may find patchy ground glass opacities, interlobar septal thickening • Laboratory Measures: • Blood cell counts • LDH • Coagulation tests • Renal function tests (creatinine, urea) • Hepatic function tests (amino function tests, bilirubin, albumin)
Management (106, 115)	First-line: • Initiation of 10 mg intravenous dexamethasone twice daily as soon as possible when DS is suspected • Continue dexamethasone until symptoms are significantly improved and taper according to institutional guidelines • Empiric treatment for other possible causes of clinical presentation (i.e., antibiotic/antiviral/antifungal agents) • Temporary discontinuation of differentiation therapy is indicated ONLY in severe, rapidly progressing cases (respiratory failure, renal failure, DIC, refractory leukocytosis) • With co-occurring leukocytosis: • Co-administration of hydroxyurea (2–4 g daily orally, titrated daily as needed) • With co-occurring tumor lysis syndrome: • Co-administration of hyperuricemia agents
Other recommendations	A WBC increase above 10 × 10^9^/L after initiating treatment with differentiation therapy should be interpreted first as DS and not immediately lead to reclassification of the patient as having high-risk disease (115).

While exact mechanisms and pathophysiology remain poorly understood, differentiation syndromes are generally thought to be inflammatory phenomena resulting from cytokine release during widespread drug-induced differentiation of immature blasts into mature cell types (51). While not yet proven in IDH-DS, it has been suggested that given its inflammatory pathogenesis, prophylactic administration of corticosteroids can reduce the incidence of retinoic acid-induced DS in APL (116). Leading mechanistic hypotheses suggest that the continuous generation of cytokines and adhesion molecules during differentiation promotes extravascular extravasation of fluid into compartments such as the lung, pleura, and pericardium in a “two-step model.” In the first step, the “initiation phase,” migration of differentiating leukemic cells is promoted by increased expression of cell surface β2 integrins and adhesion molecules such as ICAM-1 and ICAM-2—both on the surface of differentiating leukemic cells and on the pulmonary vascular endothelium. In the second step, the “aggravation phase,” there is increased production of pro-migratory chemokines by the lung and concomitant upregulation of corresponding surface receptors on the differentiating leukemic cells (117). Other studies have suggested that in addition to pulmonary infiltration of differentiating leukemic cells, there is migration of normal leukocytes into the lung as a consequence of systemically increased levels of tumor necrosis factor-alpha, interleukin (IL)-1β, IL-6, and IL-8 (118). Interestingly, a more recent study has implicated a highly-conserved nuclear protein called high-mobility group box 1 (HMGB1) in APL DS pathogenesis via the MEK/ERK signaling pathway and has suggested that HMGB1 may be a possible future target in the treatment of DS (119). Continued research is required to further elucidate the pathogenesis of DS, especially in association with the novel mutIDH inhibitors, since the overwhelming majority of existing DS research remains limited to the context of APL.

## Limitations and Potential for Combination Therapy

MutIDH inhibitors have demonstrated some efficacy in IDH-mutant AML and early results have validated their safety in glioma patients. However, given the extent of hypermethylation induced early in oncogenesis by the IDH mutation, mutIDH inhibitor monotherapy may be insufficient to thoroughly undo these preexisting and persistent global epigenetic alterations keeping the tumor cells in a dedifferentiated state. Preclinical evidence, particularly in glioma, supports the hypothesis that mutIDH assumes a “passenger,” rather than a “driver,” role soon after the development of the IDH mutation likely due to initiating methylation changes being preserved by epigenetic memory and consequent acquisition of additional driver mutations (80, 81).

The addition of hypomethylating agents, such as DNA methyltransferase (DNMT) inhibitors, to a mutIDH inhibitor regimen may generate a synergistic response by dually targeting the CIMP phenotype. In AML, it is known that patients with IDH mutations are up to 14 times more likely to response to DNMT inhibitors (120). In preclinical studies, *in vitro* human IDH1^R132H^ TF-1 erythroleukemia cells demonstrated increased cell differentiation by a factor of 25 after Ivosidenib treatment alone, which improved to a 46-fold increase with concurrent azacitidine treatment (90). Several clinical trials involving mutIDH inhibitors and DNMT inhibitors in AML are ongoing; early data was presented at the 2018 ASCO Annual Meeting from a phase 1b/2 study of combination treatment in adults with newly diagnosed IDH-mutant AML testing both Ivosidenib and Enasidenib in combination with azacitidine (ClinicalTrials.gov NCT02677922) (94). In the Ivosidenib + azacitidine group, 78% achieved a response with a 44% complete response rate, and in the Enasidenib + azacitidine group, 67% achieved a response with a 50% complete response rate. Incidence of serious adverse events were similar to those reported with azacitidine monotherapy (94). A phase 3 multicenter randomized controlled trial entitled “AGILE” will evaluate the efficacy of the Ivosidenib + azacitidine regimen compared to a placebo + azacitidine control in a similar patient population (92).

In IDH-mutant glioma, multiple DNMT inhibitors have demonstrated efficacy in preclinical studies to induce cellular differentiation, reduce global methylation and inhibit growth (121, 122). A recent study by Yamashita et al. in human IDH-mutant glioma cell lines found not only that azacitidine reduced cellular proliferation and induced increased expression of astroglial differentiation markers like GFAP, but also that azacitidine worked synergistically with temozolomide to inhibit tumor growth (123). Based on this evidence, a single phase 2 non-comparative non-randomized single center study of azacitidine in adults with recurrent IDH-mutant glioma is in the planning stages (ClinicalTrials.gov NCT03666559). However, evidence supporting combination therapy involving DNMT inhibitors and mutIDH inhibitors in glioma remains limited to early and mixed evidence from preclinical data alone (121, 123). Nevertheless, given both the synergistic response to dual therapy seen in AML and the limited efficacy of mutIDH inhibitor monotherapy in glioma trials, further investigation of combination DNMT inhibitor and mutIDH inhibitor therapy in glioma is warranted.

Epigenetic memory in IDH-mutant cancers is likely not limited to DNA methylation alone. RNA methylation patterns have also been shown to be critical for glioblastoma stem cell self-renewal and tumorigenesis specifically, and as previously discussed, the early molecular insults of IDH mutations and 2HG include competitive inhibition of histone demethylases (22, 23, 124, 125). While there is a paucity of research on RNA methylation status as a therapeutic target, histone-modifying agents such as histone deacetylase inhibitors and histone methyltransferase inhibitors are already in phase 1/2 clinical testing for AML, glioma, cholangiocarcinoma, and myelodysplastic syndromes (126–129). Early preclinical data in intrahepatic cholangiocarcinoma in particular suggests that bromodomain and extraterminal domain (BET) (histone regulatory complexes) inhibitors such as JQ1 may have an enhanced antiproliferative effect in IDH-mutant cancer cell lines (130). Furthermore, downstream oncogenic effects of histone dysregulation in IDH-mutant cancers, such as downregulation of the ATM gene causing an increase in spontaneous DNA damage, continue to become more well understood and suggest that a parallel effort to understand and target epigenetic memory carried in histones, in addition to DNA methylation, should be pursued (131).

Lastly, acquired resistance to isotype-specific treatment as a consequence of mutIDH isoform switching has recently been described as a potentially critical limitation of mutIDH inhibitor monotherapy. A small case series described two cases of IDH1^R132C^-mutant AML that, after achieving remission on Ivosidenib, both recurred with new IDH2^R140Q^ mutations (99). A third case described a similar switch from an IDH1 to IDH2 mutation in cholangiocarcinoma and a fourth case described a switch from IDH2^R140Q^-mutant AML to IDH^R132C^ after Enasidenib treatment that was then responsive to second-line treatment with pan-inhibitor, AG-881 (99). Additionally, the same research group defined another potential avenue for resistance through mutational changes in conformation of the mutIDH enzyme trans-dimer interface—the precise target of these small molecule therapeutics (132). Together, these findings suggest that despite the possibility of conversion of mutIDH from “driver” to “passenger” in supporting some chromatin modifications and cellular growth, other biological pressures for 2HG production persist over time in IDH-mutant cancers (80, 99). Future clinical studies investigating the comparative long-term efficacy of pan- and specific-mutIDH inhibition, as well as combination treatments with relevant epigenetic modifiers as previously described, may inform and improve current treatment strategies.

## Conclusions

The identification of neomorphic mutations in IDH in several cancer types including glioma and AML has generated robust research and drug discovery efforts to both elucidate critical pathways in oncogenesis and create effective, targeted molecular therapies. A host of existing mutIDH inhibitors are continually being redesigned for pharmacokinetic and pharmacodynamic optimization to permit entry into clinical trials. Two such compounds, Enasidenib (AG-221), a mutIDH2-specific inhibitor, and Ivosidenib (AG-120), a mutIDH1-specific inhibitor, have demonstrated safety and efficacy in phase 1 clinical trials for relapsed or refractory AML and recently earned FDA approval for this indication (12, 13). They are also being tested widely in other IDH-mutant cancers, such as glioma and cholangiocarcinoma (95–97). Significant early preclinical and clinical success in AML, as compared to solid tumors, may be a consequence of differences in the biological role of and oncogenic dependencies on mutIDH across these cancer subtypes. In glioma, it has been hypothesized that the mutIDH enzyme rapidly converts from an oncogenic “driver” to “passenger,” and distinctive mutIDH-associated global DNA hypermethylation patterns (CIMP) may not be readily reversible solely with mutIDH inhibition and depletion of its product, 2HG (80). In contrast, several studies in AML cellular models have demonstrated an ability for mutIDH inhibition to readily reverse DNA hypermethylation (51, 72, 74, 90). Future efforts to improve mutIDH inhibitor efficacy should focus on biologically-based combination treatment strategies, in particular with demethylating agents such as DNMT inhibitors or histone-modifying agents, which may improve response rate and duration across IDH-mutant cancers.

## Ethics Statement

In the context of this narrative review article, no consent, or IRB approval was required by the authors' institution given that no human subjects were involved in this research.

## Author Contributions

DG, NI, SD, TW, KT, and AM manuscript drafting. DG, NI, SD, TW, and DB figure and table design. DG, NI, SD, TW, DB, KT, AM, and DP manuscript editing and revision. DG, NI, SD, and TW literature review. DG, NI, and SD clinical trials review. DG, DB, KT, AM, and DP research team management and oversight.

### Conflict of Interest Statement

The authors declare that the research was conducted in the absence of any commercial or financial relationships that could be construed as a potential conflict of interest.

## References

[1] Yan H Parsons DW Jin G McLendon R Rasheed A Yuan W. IDH1 and IDH2 mutations in gliomas. N Engl J Med. (2009) 360:765–73. 10.1056/NEJMoa0808710 19228619 PMC2820383

[2] Parsons DW Jones S Zhang X Lin JC Leary RJ Angenendt P. An integrated genomic analysis of human glioblastoma multiforme. Science. (2008) 321:1807–12. 10.1126/science.1164382 18772396 PMC2820389

[3] Patel JP Gönen M Figueroa ME Fernandez H Sun Z Racevskis J. Prognostic relevance of integrated genetic profiling in acute myeloid leukemia. N Engl J Med. (2012) 366:1079–89. 10.1056/NEJMoa1112304 22417203 PMC3545649

[4] Amary MF Bacsi K Maggiani F Damato S Halai D Berisha F IDH1 and IDH2 mutations are frequent events in central chondrosarcoma and central and periosteal chondromas but not in other mesenchymal tumours. J Pathol. (2011) 224:334–43. 10.1002/path.2913 21598255

[5] Ganguly BB Kadam NN. Mutations of myelodysplastic syndromes (MDS): an update. Mutat Res Rev Mutat Res. (2016) 769:47–62. 10.1016/j.mrrev.2016.04.009 27543316

[6] Farshidfar F Zheng S Gingras MC Newton Y Shih J Robertson AG. Integrative genomic analysis of cholangiocarcinoma identifies distinct IDH-mutant molecular profiles. Cell Rep. (2017) 18:2780–94. 10.1016/j.celrep.2017.02.033 28297679 PMC5493145

[7] Johnson BE Mazor T Hong C Barnes M Aihara K McLean CY. Mutational analysis reveals the origin and therapy-driven evolution of recurrent glioma. Science. (2014) 343:189–93. 10.1126/science.1239947 24336570 PMC3998672

[8] Dombret H Itzykson R. How and when to decide between epigenetic therapy and chemotherapy in patients with AML. Hematology Am Soc Hematol Educ Program. (2017) 2017:45–53. 10.1182/asheducation-2017.1.45 29222236 PMC6142607

[9] Döhner H Estey E Grimwade D Amadori S Appelbaum FR Büchner T. Diagnosis and management of AML in adults: 2017 ELN recommendations from an international expert panel. Blood. (2017) 129:424–47. 10.1182/blood-2016-08-733196 27895058 PMC5291965

[10] Dhillon S. Ivosidenib: first global approval. Drugs. (2018) 78:1509–16. 10.1007/s40265-018-0978-3 30209701 PMC6315051

[11] Kim ES. Enasidenib: first global approval. Drugs. (2017) 77:1705–11. 10.1007/s40265-017-0813-2 28879540

[12] Stein EM DiNardo CD Pollyea DA Fathi AT Roboz GJ Altman JK Enasidenib in mutant IDH2 relapsed or refractory acute myeloid leukemia. Blood. (2017) 130:722–31. 10.1182/blood-2017-04-779405 28588020 PMC5572791

[13] DiNardo CD Stein EM de Botton S Roboz GJ Altman JK Mims AS Durable remissions with ivosidenib in IDH1-mutated relapsed or refractory AML. N Engl J Med. (2018) 378:2386–98. 10.1056/NEJMoa1716984 29860938

[14] May JL Kouri FM Hurley LA Liu J Tommasini-Ghelfi S Ji Y. IDH3alpha regulates one-carbon metabolism in glioblastoma. Sci Adv. (2019) 5:eaat0456. 10.1126/sciadv.aat0456 30613765 PMC6314828

[15] Krell D Assoku M Galloway M Mulholland P Tomlinson I Bardella C. Screen for IDH1, IDH2, IDH3, D2HGDH and L2HGDH mutations in glioblastoma. PLoS ONE. (2011) 6:e19868. 10.1371/journal.pone.0019868 21625441 PMC3100313

[16] Dang L White DW Gross S Bennett BD Bittinger MA Driggers EM. Cancer-associated IDH1 mutations produce 2-hydroxyglutarate. Nature. (2009) 462:739–44. 10.1038/nature08617 19935646 PMC2818760

[17] Bleeker FE Atai NA Lamba S Jonker A Rijkeboer D Bosch KS. The prognostic IDH1(R132) mutation is associated with reduced NADP+-dependent IDH activity in glioblastoma. Acta Neuropathol. (2010) 119:487–94. 10.1007/s00401-010-0645-6 20127344 PMC2841753

[18] Ward PS Patel J Wise DR Abdel-Wahab O Bennett BD Coller HA. The common feature of leukemia-associated IDH1 and IDH2 mutations is a neomorphic enzyme activity converting alpha-ketoglutarate to 2-hydroxyglutarate. Cancer Cell. (2010) 17:225–34. 10.1016/j.ccr.2010.01.020 20171147 PMC2849316

[19] Ward PS Lu C Cross JR Abdel-Wahab O Levine RL Schwartz GK. The potential for isocitrate dehydrogenase mutations to produce 2-hydroxyglutarate depends on allele specificity and subcellular compartmentalization. J Biol Chem. (2013) 288:3804–15. 10.1074/jbc.M112.435495 23264629 PMC3567635

[20] Brooks E Wu X Hanel A Nguyen S Wang J Zhang JH. Identification and characterization of small-molecule inhibitors of the R132H/R132H mutant isocitrate dehydrogenase 1 homodimer and R132H/wild-type heterodimer. J Biomol Screen. (2014) 19:1193–200. 10.1177/1087057114541148 24980596

[21] Madala HR Punganuru SR Arutla V Misra S Thomas TJ Srivenugopal KS. Beyond brooding on oncometabolic havoc in IDH-mutant gliomas and AML: current and future therapeutic strategies. Cancers. (2018) 10:E49. 10.3390/cancers10020049 29439493 PMC5836081

[22] Xu W Yang H Liu Y Yang Y Wang P Kim SH. Oncometabolite 2-hydroxyglutarate is a competitive inhibitor of alpha-ketoglutarate-dependent dioxygenases. Cancer Cell. (2011) 19:17–30. 10.1016/j.ccr.2010.12.014 21251613 PMC3229304

[23] Clark O Yen K Mellinghoff IK. Molecular pathways: isocitrate dehydrogenase mutations in cancer. Clin Cancer Res. (2016) 22:1837–42. 10.1158/1078-0432.CCR-13-1333 26819452 PMC4834266

[24] Clifton IJ McDonough MA Ehrismann D Kershaw NJ Granatino N Schofield CJ. Structural studies on 2-oxoglutarate oxygenases and related double-stranded beta-helix fold proteins. J Inorg Biochem. (2006) 100:644–69. 10.1016/j.jinorgbio.2006.01.024 16513174

[25] Chowdhury R Yeoh KK Tian YM Hillringhaus L Bagg EA Rose NR. The oncometabolite 2-hydroxyglutarate inhibits histone lysine demethylases. EMBO Rep. (2011) 12:463–9. 10.1038/embor.2011.43 21460794 PMC3090014

[26] Joberty G Boesche M Brown JA Eberhard D Garton NS Humphreys PG. Interrogating the druggability of the 2-oxoglutarate-dependent dioxygenase target class by chemical proteomics. ACS Chem Biol. (2016) 11:2002–10. 10.1021/acschembio.6b00080 27197014

[27] Turcan S Makarov V Taranda J Wang Y Fabius AWM Wu W. Mutant-IDH1-dependent chromatin state reprogramming, reversibility, and persistence. Nat Genet. (2018) 50:62–72. 10.1038/s41588-017-0001-z 29180699 PMC5769471

[28] Malta TM de Souza CF Sabedot TS Silva TC Mosella MS Kalkanis SN. Glioma CpG island methylator phenotype (G-CIMP): biological and clinical implications. Neuro Oncol. (2018) 20:608–20. 10.1093/neuonc/nox183 29036500 PMC5892155

[29] Turcan S Rohle D Goenka A Walsh LA Fang F Yilmaz E. IDH1 mutation is sufficient to establish the glioma hypermethylator phenotype. Nature. (2012) 483:479–83. 10.1038/nature10866 22343889 PMC3351699

[30] Morita R Hirohashi Y Suzuki H Takahashi A Tamura Y Kanaseki T. DNA methyltransferase 1 is essential for initiation of the colon cancers. Exp Mol Pathol. (2013) 94:322–9. 10.1016/j.yexmp.2012.10.004 23064049

[31] Wongtrakoongate P. Epigenetic therapy of cancer stem and progenitor cells by targeting DNA methylation machineries. World J Stem Cells. (2015) 7:137–48. 10.4252/wjsc.v7.i1.137 25621113 PMC4300924

[32] Dunn GP Rinne ML Wykosky J Genovese G Quayle SN Dunn IF. Emerging insights into the molecular and cellular basis of glioblastoma. Genes Dev. (2012) 26:756–84. 10.1101/gad.187922.112 22508724 PMC3337451

[33] Yan H Bigner DD Velculescu V Parsons DW. Mutant metabolic enzymes are at the origin of gliomas. Cancer Res. (2009) 69:9157–9. 10.1158/0008-5472.CAN-09-2650 19996293 PMC2794981

[34] Bleeker FE Lamba S Leenstra S Troost D Hulsebos T Vandertop WP IDH1 mutations at residue p.R132 (IDH1(R132)) occur frequently in high-grade gliomas but not in other solid tumors. Hum Mutat. (2009) 30:7–11. 10.1002/humu.20937 19117336

[35] Hartmann C Meyer J Balss J Capper D Mueller W Christians A. Type and frequency of IDH1 and IDH2 mutations are related to astrocytic and oligodendroglial differentiation and age: a study of 1,010 diffuse gliomas. Acta Neuropathol. (2009) 118:469–74. 10.1007/s00401-009-0561-9 19554337

[36] Koh J Cho H Kim H Kim SI Yun S Park CK. IDH2 mutation in gliomas including novel mutation. Neuropathology. (2015) 35:236–44. 10.1111/neup.12187 25495392

[37] Cancer Genome Atlas Research Network Brat DJ Verhaak RG Aldape KD Yung WK Salama SR Comprehensive, integrative genomic analysis of diffuse lower-grade gliomas. N Engl J Med. (2015) 372:2481–98. 10.1056/NEJMoa1402121 26061751 PMC4530011

[38] Flavahan WA Drier Y Liau BB Gillespie SM Venteicher AS Stemmer-Rachamimov AO. Insulator dysfunction and oncogene activation in IDH mutant gliomas. Nature. (2016) 529:110–4. 10.1038/nature16490 26700815 PMC4831574

[39] Modrek AS Golub D Khan T Bready D Prado J Bowman C. Low-grade astrocytoma mutations in IDH1, P53, and ATRX cooperate to block differentiation of human neural stem cells via repression of SOX2. Cell Rep. (2017) 21:1267–80. 10.1016/j.celrep.2017.10.009 29091765 PMC5687844

[40] Watanabe T Nobusawa S Kleihues P Ohgaki H. IDH1 mutations are early events in the development of astrocytomas and oligodendrogliomas. Am J Pathol. (2009) 174:1149–53. 10.2353/ajpath.2009.080958 19246647 PMC2671348

[41] Bardella C Al-Dalahmah O Krell D Brazauskas P Al-Qahtani K Tomkova M Expression of Idh1(R132H) in the murine subventricular zone stem cell niche recapitulates features of early gliomagenesis. Cancer Cell. (2016) 30:578–94. 10.1016/j.ccell.2016.08.017 27693047 PMC5064912

[42] Pirozzi CJ Carpenter AB Waitkus MS Wang CY Zhu H Hansen LJ. Mutant IDH1 disrupts the mouse subventricular zone and alters brain tumor progression. Mol Cancer Res. (2017) 15:507–20. 10.1158/1541-7786.MCR-16-0485 28148827 PMC5415422

[43] Mardis ER Ding L Dooling DJ Larson DE McLellan MD Chen K. Recurring mutations found by sequencing an acute myeloid leukemia genome. N Engl J Med. (2009) 361:1058–66. 10.1056/NEJMoa0903840 19657110 PMC3201812

[44] Dohner H Weisdorf DJ Bloomfield CD. Acute myeloid leukemia. N Engl J Med. (2015) 373:1136–52. 10.1056/NEJMra1406184 26376137

[45] DiNardo CD Ravandi F Agresta S Konopleva M Takahashi K Kadia T. Characteristics, clinical outcome, and prognostic significance of IDH mutations in AML. Am J Hematol. (2015) 90:732–6. 10.1002/ajh.24072 26016821 PMC4612499

[46] Im AP Sehgal AR Carroll MP Smith BD Tefferi A Johnson DE. DNMT3A and IDH mutations in acute myeloid leukemia and other myeloid malignancies: associations with prognosis and potential treatment strategies. Leukemia. (2014) 28:1774–83. 10.1038/leu.2014.124 24699305 PMC4234093

[47] Marcucci G Maharry K Wu YZ Radmacher MD Mrózek K Margeson D. IDH1 and IDH2 gene mutations identify novel molecular subsets within *de novo* cytogenetically normal acute myeloid leukemia: a Cancer and Leukemia Group B study. J Clin Oncol. (2010) 28:2348–55. 10.1200/JCO.2009.27.3730 20368543 PMC2881719

[48] Wagner K Damm F Göhring G Görlich K Heuser M Schäfer I. Impact of IDH1 R132 mutations and an IDH1 single nucleotide polymorphism in cytogenetically normal acute myeloid leukemia: SNP rs11554137 is an adverse prognostic factor. J Clin Oncol. (2010) 28:2356–64. 10.1200/JCO.2009.27.6899 20368538

[49] Thol F Damm F Wagner K Göhring G Schlegelberger B Hoelzer D. Prognostic impact of IDH2 mutations in cytogenetically normal acute myeloid leukemia. Blood. (2010) 116:614–6. 10.1182/blood-2010-03-272146 20421455

[50] Thomas D Majeti R. Optimizing next-generation AML therapy: activity of mutant IDH2 inhibitor AG-221 in preclinical models. Cancer Discov. (2017) 7:459–61. 10.1158/2159-8290.CD-17-0270 28461409 PMC5456121

[51] Yen K Travins J Wang F David MD Artin E Straley K. AG-221, a first-in-class therapy targeting acute myeloid leukemia harboring oncogenic IDH2 mutations. Cancer Discov. (2017) 7:478–93. 10.1158/2159-8290.CD-16-1034 28193778

[52] Chaturvedi A Herbst L Pusch S Klett L Goparaju R Stichel D. Pan-mutant-IDH1 inhibitor BAY1436032 is highly effective against human IDH1 mutant acute myeloid leukemia *in vivo*. Leukemia. (2017) 31:2020–8. 10.1038/leu.2017.46 28232670 PMC5629366

[53] Ho PA Kopecky KJ Alonzo TA Gerbing RB Miller KL Kuhn J. Prognostic implications of the IDH1 synonymous SNP rs11554137 in pediatric and adult AML: a report from the Children's Oncology Group and SWOG. Blood. (2011) 118:4561–6. 10.1182/blood-2011-04-348888 21873548 PMC3208275

[54] Mondesir J Willekens C Touat M de Botton S. IDH1 and IDH2 mutations as novel therapeutic targets: current perspectives. J Blood Med. (2016) 7:171–80. 10.2147/JBM.S70716 27621679 PMC5015873

[55] Medeiros BC Fathi AT DiNardo CD Pollyea DA Chan SM Swords R. Isocitrate dehydrogenase mutations in myeloid malignancies. Leukemia. (2017) 31:272–81. 10.1038/leu.2016.275 27721426 PMC5292675

[56] Green CL Evans CM Hills RK Burnett AK Linch DC Gale RE. The prognostic significance of IDH1 mutations in younger adult patients with acute myeloid leukemia is dependent on FLT3/ITD status. Blood. (2010) 116:2779–82. 10.1182/blood-2010-02-270926 20651067

[57] Green CL Evans CM Zhao L Hills RK Burnett AK Linch DC. The prognostic significance of IDH2 mutations in AML depends on the location of the mutation. Blood. (2011) 118:409–12. 10.1182/blood-2010-12-322479 21596855

[58] DiNardo CD Propert KJ Loren AW Paietta E Sun Z Levine RL. Serum 2-hydroxyglutarate levels predict isocitrate dehydrogenase mutations and clinical outcome in acute myeloid leukemia. Blood. (2013) 121:4917–24. 10.1182/blood-2013-03-493197 23641016 PMC3682342

[59] Wang JH Chen WL Li JM Wu SF Chen TL Zhu YM. Prognostic significance of 2-hydroxyglutarate levels in acute myeloid leukemia in China. Proc Natl Acad Sci USA. (2013) 110:17017–22. 10.1073/pnas.1315558110 24082129 PMC3801077

[60] Chen J Yang J Cao P. The evolving landscape in the development of isocitrate dehydrogenase mutant inhibitors. Mini Rev Med Chem. (2016) 16:1344–58. 10.2174/1389557516666160609085520 27292784

[61] Ma R Yun CH. Crystal structures of pan-IDH inhibitor AG-881 in complex with mutant human IDH1 and IDH2. Biochem Biophys Res Commun. (2018) 503:2912–7. 10.1016/j.bbrc.2018.08.068 30131249

[62] Yen K Konteatis Z Sui Z Artin E Dang L Straley K Abstract B126: AG-881, a brain penetrant, potent, pan-mutant IDH (mIDH) inhibitor for use in mIDH solid and hematologic malignancies. Mol Cancer Ther. (2018) 17(suppl. 1):B126 10.1158/1535-7163.TARG-17-B126

[63] Pusch S Krausert S Fischer V Balss J Ott M Schrimpf D. Pan-mutant IDH1 inhibitor BAY 1436032 for effective treatment of IDH1 mutant astrocytoma *in vivo*. Acta Neuropathol. (2017) 133:629–44. 10.1007/s00401-017-1677-y 28124097

[64] DiNardo CD Stein EM. SOHO state of the art update and next questions: IDH therapeutic targeting in AML. Clinical Lymphoma Myeloma Leuk. (2018) 18:769–72. 10.1016/j.clml.2018.10.007 30416011

[65] Corporation C Celgene Corporation and Agios Pharmaceuticals Announce Global Strategic Collaboration to Advance Unique Science of Cancer Metabolism [Media Release]. (2010).

[66] Popovici-Muller J Saunders JO Salituro FG Travins JM Yan S Zhao F. Discovery of the first potent inhibitors of mutant IDH1 that lower tumor 2-HG *in vivo*. ACS Med Chem Lett. (2012) 3:850–5. 10.1021/ml300225h 24900389 PMC4025665

[67] Rohle D Popovici-Muller J Palaskas N Turcan S Grommes C Campos C. An inhibitor of mutant IDH1 delays growth and promotes differentiation of glioma cells. Science. (2013) 340:626–30. 10.1126/science.1236062 23558169 PMC3985613

[68] Popovici-Muller J Lemieux RM Artin E Saunders JO Salituro FG Travins J. Discovery of AG-120 (Ivosidenib): a first-in-class mutant IDH1 inhibitor for the treatment of IDH1 mutant cancers. ACS Med Chem Lett. (2018) 9:300–5. 10.1021/acsmedchemlett.7b00421 29670690 PMC5900343

[69] Mellinghoff I Penas-Prado M Peters K Cloughesy T Burris H Maher E ACTR-31. Phase 1 study of AG-881, an inhibitor of mutant IDH1 and IDH2: results from the recurrent/progressive glioma population. Neuro Oncol. (2018) 20(suppl. 6):vi18 10.1093/neuonc/noy148.064

[70] Li L Paz AC Wilky BA Johnson B Galoian K Rosenberg A. Treatment with a small molecule mutant IDH1 inhibitor suppresses tumorigenic activity and decreases production of the oncometabolite 2-hydroxyglutarate in human chondrosarcoma cells. PLoS ONE. (2015) 10:e0133813. 10.1371/journal.pone.0133813 26368816 PMC4569544

[71] Cho YS Levell JR Liu G Caferro T Sutton J Shafer CM. Discovery and evaluation of clinical candidate IDH305, a brain penetrant mutant IDH1 inhibitor. ACS Med Chem Lett. (2017) 8:1116–21. 10.1021/acsmedchemlett.7b00342 29057061 PMC5641959

[72] Wang F Travins J DeLaBarre B Penard-Lacronique V Schalm S Hansen E. Targeted inhibition of mutant IDH2 in leukemia cells induces cellular differentiation. Science. (2013) 340:622–6. 10.1126/science.1234769 23558173

[73] Chen J Yang J Sun X Wang Z Cheng X Lu W. Allosteric inhibitor remotely modulates the conformation of the orthestric pockets in mutant IDH2/R140Q. Sci Rep. (2017) 7:16458. 10.1038/s41598-017-16427-w 29184081 PMC5705638

[74] Kernytsky A Wang F Hansen E Schalm S Straley K Gliser C. IDH2 mutation-induced histone and DNA hypermethylation is progressively reversed by small-molecule inhibition. Blood. (2015) 125:296–303. 10.1182/blood-2013-10-533604 25398940 PMC4295919

[75] Watts JM Baer MR Lee S Yang J Dinner SN Prebet T A phase 1 dose escalation study of the IDH1m inhibitor, FT-2102, in patients with acute myeloid leukemia (AML) or myelodysplastic syndrome (MDS). J Clin Oncol. (2018) 36(suppl. 15):7009 10.1200/JCO.2018.36.15_suppl.7009

[76] Chaturvedi A Araujo Cruz MM Jyotsana N Sharma A Yun H Görlich K. Mutant IDH1 promotes leukemogenesis *in vivo* and can be specifically targeted in human AML. Blood. (2013) 122:2877–87. 10.1182/blood-2013-03-491571 23954893

[77] Chaturvedi A Araujo Cruz MM Goparaju R Jyotsana N Baehre H Goerlich K A novel inhibitor of mutant IDH1 induces differentiation *in vivo* and prolongs survival in a mouse model of leukemia. Blood. (2014) 124:3598 Available online at: http://www.bloodjournal.org/content/124/21/3598/tab-article-info

[78] Kopinja J Sevilla RS Levitan D Dai D Vanko A Spooner E. A brain penetrant mutant IDH1 inhibitor provides *in vivo* survival benefit. Sci Rep. (2017) 7:13853. 10.1038/s41598-017-14065-w 29062039 PMC5653818

[79] Okoye-Okafor UC Bartholdy B Cartier J Gao EN Pietrak B Rendina AR. New IDH1 mutant inhibitors for treatment of acute myeloid leukemia. Nat Chem Biol. (2015) 11:878–86. 10.1038/nchembio.1930 26436839 PMC5155016

[80] Johannessen TA Mukherjee J Viswanath P Ohba S Ronen SM Bjerkvig R. Rapid conversion of mutant IDH1 from driver to passenger in a model of human gliomagenesis. Mol Cancer Res. (2016) 14:976–83. 10.1158/1541-7786.MCR-16-0141 27430238 PMC5065766

[81] Tateishi K Wakimoto H Iafrate AJ Tanaka S Loebel F Lelic N. Extreme vulnerability of IDH1 mutant cancers to NAD+ depletion. Cancer Cell. (2015) 28:773–84. 10.1016/j.ccell.2015.11.006 26678339 PMC4684594

[82] Levell JR Caferro T Chenail G Dix I Dooley J Firestone B. Optimization of 3-pyrimidin-4-yl-oxazolidin-2-ones as allosteric and mutant specific inhibitors of IDH1. ACS Med Chem Lett. (2017) 8:151–6. 10.1021/acsmedchemlett.6b00334 28197303 PMC5304300

[83] DiNardo CD Schimmer AD Yee KWL A Phase I study of IDH305 in patients with advanced malignancies including relapsed/refractory AML and MDS that harbor IDH1R132 Mutations. Blood. (2016) 128:1073 Available online at: http://www.bloodjournal.org/content/128/22/1073

[84] Calvert AE Chalastanis A Wu Y Hurley LA Kouri FM Bi Y. Cancer-associated IDH1 promotes growth and resistance to targeted therapies in the absence of mutation. Cell Rep. (2017) 19:1858–73. 10.1016/j.celrep.2017.05.014 28564604 PMC5564207

[85] Yang B Zhong C Peng Y Lai Z Ding J Molecular mechanisms of “off-on switch” of activities of human IDH1 by tumor-associated mutation R132H. *Cell Res*. (2010) 20:1188–200. 10.1038/cr.2010.145 20975740

[86] Stein EM Enasidenib, a targeted inhibitor of mutant IDH2 proteins for treatment of relapsed or refractory acute myeloid leukemia. Future Oncol. (2018) 14:23–40. 10.2217/fon-2017-0392 29243965

[87] Deng G Shen J Yin M McManus J Mathieu M Gee P. Selective inhibition of mutant isocitrate dehydrogenase 1 (IDH1) via disruption of a metal binding network by an allosteric small molecule. J Biol Chem. (2015) 290:762–74. 10.1074/jbc.M114.608497 25391653 PMC4294499

[88] Heredia V 1524P - AG-120, a novel IDH1 targeted molecule, inhibits invasion and migration of chondrosarcoma cells *in vitro*. Ann Oncol. (2017) 28:v521–38. 10.1093/annonc/mdx387.049

[89] Nicolay B Narayanaswamy R Aguado E Nagaraja R Murtie J Liu G EXTH-59. The IDH1 mutant inhibitor AG-120 shows strong inhibition of 2-HG production in an orthotopic IDH1 mutant glioma model *in vivo*. Neuro Oncol. (2017) 19(suppl. 6):vi86 10.1093/neuonc/nox168.351

[90] Yen K Chopra VS Tobin E Avanzino B Mavrommatis K Dimartino J Abstract 4956: functional characterization of the ivosidenib (AG-120) and azacitidine combination in a mutant IDH1 AML cell model. Cancer Res. (2018) 78(suppl. 13):4956 10.1158/1538-7445.AM2018-4956

[91] Tallman MS Knight RD Glasmacher AG Dohner H Phase III randomized, open-label study comparing the efficacy and safety of AG-221 vs conventional care regimens (CCR) in older patients with advanced acute myeloid leukemia (AML) with isocitrate dehydrogenase (IDH)-2 mutations in relapse or refractory to multiple prior treatments: the idhentify trial. J Clin Oncol. (2016) 34(suppl. 15):TPS7074 10.1200/JCO.2016.34.15_suppl.TPS7074

[92] Stein E Dinardo CD Jang JH Miyazaki Y Martinez RO Auer J AGILE: A phase 3, multicenter, randomized, placebo-controlled study of ivosidenib in combination with azacitidine in adult patients with previously untreated acute myeloid leukemia with an IDH1 mutation. J Clin Oncol. (2018) 36(suppl. 15):TPS7074 10.1200/JCO.2018.36.15_suppl.TPS7074

[93] Stein EM DiNardo CD Mims AS Savona MR Pratz K Stein AS. Ivosidenib or enasidenib combined with standard induction chemotherapy is well tolerated and active in patients with newly diagnosed AML with an IDH1 or IDH2 mutation: initial results from a Phase 1 trial. Blood. (2017) 130(suppl. 1):726. Available online at: http://www.bloodjournal.org/content/130/Suppl_1/726/tab-article-info30668823

[94] Dinardo CD Stein AS Stein EM Fathi AT Montesinos P Odenike O Mutant IDH (mIDH) inhibitors, ivosidenib or enasidenib, with azacitidine (AZA) in patients with acute myeloid leukemia (AML). J Clin Oncol. (2018) 36(suppl. 15):7042 10.1200/JCO.2018.36.15_suppl.7042

[95] Burris H Mellinghoff I Maher E Wen P Beeram M Touat M Abstract PL04–05: the first reported results of AG-120, a first-in-class, potent inhibitor of the IDH1 mutant protein, in a Phase I study of patients with advanced IDH1-mutant solid tumors, including gliomas. Mol Cancer Ther. (2015) 14(suppl. 2):PL04–05. 10.1158/1535-7163.TARG-15-PL04-05

[96] Mellinghoff IK Touat M Maher E DeLaFuente M Cloughesy TF Holdhoff M ACTR-46. AG120, A first-in-class mutant IDH1 inhibitor in patients with recurrent or progressive IDH1 mutant glioma: results from the Phase 1 glioma expansion cohorts. Neuro Oncol. (2016) 18(suppl. 6):vi12 10.1093/neuonc/now212.044

[97] Fan B Mellinghoff IK Wen PY Lowery MA Goyal L Tap WD Pharmacokinetics/pharmacodynamics (PK/PD) of ivosidenib in patients with IDH1-mutant advanced solid tumors from a phase 1 study. J Clin Oncol. (2018) 36(suppl. 15):2577 10.1200/JCO.2018.36.15_suppl.2577

[98] Mellinghoff IK Penas-Prado M Peters KB Cloughesy TF Burris HA Burris EA Phase 1 study of AG-881, an inhibitor of mutant IDH1/IDH2, in patients with advanced IDH-mutant solid tumors, including glioma. J Clin Oncol. (2018) 36(suppl. 15):2002 10.1200/JCO.2018.36.15_suppl.200229746224

[99] Harding JJ Lowery MA Shih AH Schvartzman JM Hou S Famulare C. Isoform switching as a mechanism of acquired resistance to mutant isocitrate dehydrogenase inhibition. Cancer Discov. (2018) 8:1540–7. 10.1158/2159-8290.CD-18-0877 30355724 PMC6699636

[100] Lowery MA Abou-Alfa GK Valle JW Kelley RK Goyal L Shroff RT ClarIDHy: a phase 3, multicenter, randomized, double-blind study of AG-120 vs placebo in patients with an advanced cholangiocarcinoma with an IDH1 mutation. J Clin Oncol. (2017) 35(suppl. 15):TPS4142 10.1200/JCO.2017.35.15_suppl.TPS4142

[101] Mellinghoff I Le K Young R Maher E Wen P Cloughesy T RBTT-03. A phase 1, multicenter, randomized, open-label, perioperative study of AG-120 (ivosidenib) and AG-881 in patients with recurrent, nonenhancing, IDH1-mutant, low-grade glioma. Neuro Oncol. (2018) 20(suppl. 6):vi234 10.1093/neuonc/noy148.973

[102] Lowery MA Abou-Alfa GK Burris HA Janku F Shroff RT Cleary JM Phase I study of AG-120, an IDH1 mutant enzyme inhibitor: results from the cholangiocarcinoma dose escalation and expansion cohorts. J Clin Oncol. (2017) 35(15_suppl):4015 10.1200/JCO.2017.35.15_suppl.4015

[103] Frankel SR Eardley A Lauwers G Weiss M Warrell RP Jr The “retinoic acid syndrome” in acute promyelocytic leukemia. Ann Intern Med. (1992) 117:292–6. 10.7326/0003-4819-117-4-292 1637024

[104] Au WY Kwong YL. Arsenic trioxide: safety issues and their management. Acta Pharmacol Sin. (2008) 29:296–304. 10.1111/j.1745-7254.2008.00771.x 18298894

[105] Fathi AT DiNardo CD Kline I Kenvin L Gupta I Attar EC. Differentiation syndrome associated with enasidenib, a selective inhibitor of mutant isocitrate dehydrogenase 2: analysis of a phase 1/2 study. JAMA Oncol. (2018) 4:1106–10. 10.1001/jamaoncol.2017.4695 29346478 PMC5885269

[106] Birendra KC DiNardo CD. Evidence for clinical differentiation and differentiation syndrome in patients with acute myeloid leukemia and IDH1 mutations treated with the targeted mutant IDH1 inhibitor, AG-120. Clin Lymphoma Myeloma Leuk. (2016) 16:460–5. 10.1016/j.clml.2016.04.006 27245312 PMC4983480

[107] Mascarenhas J. A concise update on risk factors, therapy, and outcome of leukemic transformation of myeloproliferative neoplasms. Clin Lymphoma Myeloma Leuk. (2016) 16:S124–9. 10.1016/j.clml.2016.02.016 27521308

[108] Yogarajah M Tefferi A. Leukemic transformation in myeloproliferative neoplasms: a literature review on risk, characteristics, and outcome. Mayo Clin Proc. (2017) 92:1118–28. 10.1016/j.mayocp.2017.05.010 28688466

[109] Daly PA Schiffer CA Wiernik PH. Acute promyelocytic leukemia–clinical management of 15 patients. Am J Hematol. (1980) 8:347–59. 10.1002/ajh.2830080403 6932177

[110] Leblebjian H DeAngelo DJ Skirvin JA Stone RM Wadleigh M Werner L. Predictive factors for all-trans retinoic acid-related differentiation syndrome in patients with acute promyelocytic leukemia. Leuk Res. (2013) 37:747–51. 10.1016/j.leukres.2013.04.011 23643326

[111] Jeddi R Ghedira H Mnif S Gouider E Fenaux P Meddeb B. High body mass index is an independent predictor of differentiation syndrome in patients with acute promyelocytic leukemia. Leuk Res. (2010) 34:545–7. 10.1016/j.leukres.2009.09.017 19800119

[112] Montesinos P Bergua JM Vellenga E Rayón C Parody R de la Serna J. Differentiation syndrome in patients with acute promyelocytic leukemia treated with all-trans retinoic acid and anthracycline chemotherapy: characteristics, outcome, and prognostic factors. Blood. (2009) 113:775–83. 10.1182/blood-2008-07-168617 18945964

[113] Elemam O Abdelmoety D. Acute promyelocytic leukemia, study of predictive factors for differentiation syndrome, single center experience. J Egypt Natl Cancer Inst. (2013) 25:13–9. 10.1016/j.jnci.2012.10.004 23499202

[114] Cardinale L Asteggiano F Moretti F Torre F Ulisciani S Fava C. Pathophysiology, clinical features and radiological findings of differentiation syndrome/all-trans-retinoic acid syndrome. World J Radiol. (2014) 6:583–8. 10.4329/wjr.v6.i8.583 25170395 PMC4147438

[115] Sanz MA Fenaux P Tallman MS Estey EH Löwenberg B Naoe T. Management of acute promyelocytic leukemia: updated recommendations from an expert panel of the European LeukemiaNet. Blood. (2019) 133:1630–43. 10.1182/blood-2019-01-894980 30803991 PMC6509567

[116] Montesinos P Sanz MA. The differentiation syndrome in patients with acute promyelocytic leukemia: experience of the pethema group and review of the literature. Mediterr J Hematol Infect Dis. (2011) 3:e2011059. 10.4084/mjhid.2011.059 22220256 PMC3248336

[117] Luesink M Jansen JH. Advances in understanding the pulmonary infiltration in acute promyelocytic leukaemia. Br J Haematol. (2010) 151:209–20. 10.1111/j.1365-2141.2010.08325.x 20735400

[118] De Botton S Dombret H Sanz M Miguel JS Caillot D Zittoun R Incidence, clinical features, and outcome of all trans-retinoic acid syndrome in 413 cases of newly diagnosed acute promyelocytic leukemia. The European APL Group. Blood. (1998) 92:2712–8. 9763554

[119] Tang L Chai W Ye F Yu Y Cao L Yang M. HMGB1 promotes differentiation syndrome by inducing hyperinflammation via MEK/ERK signaling in acute promyelocytic leukemia cells. Oncotarget. (2017) 8:27314–27. 10.18632/oncotarget.15432 28404891 PMC5432337

[120] Emadi A Faramand R Carter-Cooper B Tolu S Ford LA Lapidus RG. Presence of isocitrate dehydrogenase mutations may predict clinical response to hypomethylating agents in patients with acute myeloid leukemia. Am J Hematol. (2015) 90:E77–9. 10.1002/ajh.23965 25651001

[121] Turcan S Fabius AW Borodovsky A Pedraza A Brennan C Huse J. Efficient induction of differentiation and growth inhibition in IDH1 mutant glioma cells by the DNMT Inhibitor Decitabine. Oncotarget. (2013) 4:1729–36. 10.18632/oncotarget.1412 24077826 PMC3858559

[122] Borodovsky A Salmasi V Turcan S Fabius AW Baia GS Eberhart CG. 5-azacytidine reduces methylation, promotes differentiation and induces tumor regression in a patient-derived IDH1 mutant glioma xenograft. Oncotarget. (2013) 4:1737–47. 10.18632/oncotarget.1408 24077805 PMC3858560

[123] Yamashita AS da Costa Rosa M Borodovsky A Festuccia WT Chan T Riggins GJ Demethylation and epigenetic modification with 5-azacytidine reduces IDH1 mutant glioma growth in combination with temozolomide. Neuro Oncol. (2018) 21:189–200. 10.1093/neuonc/noy146 PMC6374765 30184215

[124] Dixit D Xie Q Rich JN Zhao JC. Messenger RNA methylation regulates glioblastoma tumorigenesis. Cancer Cell. (2017) 31:474–5. 10.1016/j.ccell.2017.03.010 28399407 PMC6482444

[125] Cui Q Shi H Ye P Li L Qu Q Sun G m(6)A RNA methylation regulates the self-renewal and tumorigenesis of glioblastoma stem cells. Cell Rep. (2017) 18:2622–34. 10.1016/j.celrep.2017.02.059 28297667 PMC5479356

[126] Williams MJ Singleton WG Lowis SP Malik K Kurian KM. Therapeutic targeting of histone modifications in adult and pediatric high-grade glioma. Front Oncol. (2017) 7:45. 10.3389/fonc.2017.00045 28401060 PMC5368219

[127] Lu R Wang GG. Pharmacologic targeting of chromatin modulators as therapeutics of acute myeloid leukemia. Front Oncol. (2017) 7:241. 10.3389/fonc.2017.00241 29075615 PMC5643408

[128] Ungerstedt JS. Epigenetic modifiers in myeloid malignancies: the role of histone deacetylase inhibitors. Int J Mol Sci. (2018) 19:E3091. 10.3390/ijms19103091 30304859 PMC6212943

[129] Moeini A Sia D Bardeesy N Mazzaferro V Llovet JM. Molecular pathogenesis and targeted therapies for intrahepatic cholangiocarcinoma. Clin Cancer Res. (2016) 22:291–300. 10.1158/1078-0432.CCR-14-3296 26405193 PMC12224570

[130] Fujiwara H Tateishi K Kato H Nakatsuka T Yamamoto K Tanaka Y. Isocitrate dehydrogenase 1 mutation sensitizes intrahepatic cholangiocarcinoma to the BET inhibitor JQ1. Cancer Sci. (2018) 109:3602–10. 10.1111/cas.13784 30156013 PMC6215870

[131] Inoue S Li WY Tseng A Beerman I Elia AJ Bendall SC. Mutant IDH1 downregulates ATM and alters DNA repair and sensitivity to DNA damage independent of TET2. Cancer Cell. (2016) 30:337–48. 10.1016/j.ccell.2016.05.018 27424808 PMC5022794

[132] Intlekofer AM Shih AH Wang B Nazir A Rustenburg AS Albanese SK. Acquired resistance to IDH inhibition through trans or cis dimer-interface mutations. Nature. (2018) 559:125–9. 10.1038/s41586-018-0251-7 29950729 PMC6121718

